# Genetic and phenotypic characterization of complex hereditary spastic paraplegia

**DOI:** 10.1093/brain/aww111

**Published:** 2016-05-23

**Authors:** Eleanna Kara, Arianna Tucci, Claudia Manzoni, David S. Lynch, Marilena Elpidorou, Conceicao Bettencourt, Viorica Chelban, Andreea Manole, Sherifa A. Hamed, Nourelhoda A. Haridy, Monica Federoff, Elisavet Preza, Deborah Hughes, Alan Pittman, Zane Jaunmuktane, Sebastian Brandner, Georgia Xiromerisiou, Sarah Wiethoff, Lucia Schottlaender, Christos Proukakis, Huw Morris, Tom Warner, Kailash P. Bhatia, L.V. Prasad Korlipara, Andrew B. Singleton, John Hardy, Nicholas W. Wood, Patrick A. Lewis, Henry Houlden

**Affiliations:** ^1^ 1 Department of Molecular Neuroscience, UCL Institute of Neurology, Queen Square, London WC1N 3BG, UK; ^2^ 2 Alzheimer’s Disease Research Centre, Department of Neurology, Harvard Medical School and Massachusetts General Hospital, 114 16th Street, Charlestown, MA 02129, USA; ^3^ 3 Department of Pathophysiology and Transplantation, Università degli Studi di Milano, Milano, Italy; ^4^ 4 School of Pharmacy, University of Reading, Reading RG6 6AP, UK; ^5^ 5 Department of Neurology and Psychiatry, Assiut University Hospital, Faculty of Medicine, Assiut, Egypt; ^6^ 6 Laboratory of Neurogenetics, NIH/NIA, Bethesda, MD 20892, USA; ^7^ 7 Division of Neuropathology and Department of Neurodegenerative Disease, The National Hospital for Neurology and Neurosurgery, UCL Institute of Neurology, Queen Square, London WC1N 3BG, UK; ^8^ 8 Department of Neurology, Papageorgiou Hospital, Thessaloniki, Greece; ^9^ 9 Department of Clinical Neuroscience, Royal Free Campus, UCL Institute of Neurology, London, UK; ^10^ 10 Reta Lila Weston Institute of Neurological Studies and Queen Square Brain Bank for Neurological Disorders, UCL Institute of Neurology, Queen Square, London WC1N 3BG, UK; ^11^ 11 Sobell Department of Motor Neuroscience and Movement Disorders, UCL Institute of Neurology, Queen Square, London, WC1N 3BG, UK; ^12^ 12 Neurogenetics Laboratory, The National Hospital for Neurology and Neurosurgery, Queen Square, London WC1N 3BG, UK

**Keywords:** hereditary spastic paraplegia, *SPG11*, gene, mutation, Parkinson’s disease

## Abstract

The hereditary spastic paraplegias are a heterogeneous group of degenerative disorders that are clinically classified as either pure with predominant lower limb spasticity, or complex where spastic paraplegia is complicated with additional neurological features, and are inherited in autosomal dominant, autosomal recessive or X-linked patterns. Genetic defects have been identified in over 40 different genes, with more than 70 loci in total. Complex recessive spastic paraplegias have in the past been frequently associated with mutations in *SPG11* (spatacsin), *ZFYVE26/SPG15* , *SPG7* (paraplegin) and a handful of other rare genes, but many cases remain genetically undefined. The overlap with other neurodegenerative disorders has been implied in a small number of reports, but not in larger disease series. This deficiency has been largely due to the lack of suitable high throughput techniques to investigate the genetic basis of disease, but the recent availability of next generation sequencing can facilitate the identification of disease-causing mutations even in extremely heterogeneous disorders. We investigated a series of 97 index cases with complex spastic paraplegia referred to a tertiary referral neurology centre in London for diagnosis or management. The mean age of onset was 16 years (range 3 to 39). The *SPG11* gene was first analysed, revealing homozygous or compound heterozygous mutations in 30/97 (30.9%) of probands, the largest *SPG11* series reported to date, and by far the most common cause of complex spastic paraplegia in the UK, with severe and progressive clinical features and other neurological manifestations, linked with magnetic resonance imaging defects. Given the high frequency of *SPG11* mutations, we studied the autophagic response to starvation in eight affected *SPG11* cases and control fibroblast cell lines, but in our restricted study we did not observe correlations between disease status and autophagic or lysosomal markers. In the remaining cases, next generation sequencing was carried out revealing variants in a number of other known complex spastic paraplegia genes, including five in *SPG7* (5/97), four in *FA2H* (also known as *SPG35* ) (4/97) and two in *ZFYVE26* / *SPG15* . Variants were identified in genes usually associated with pure spastic paraplegia and also in the Parkinson’s disease-associated gene *ATP13A2* , neuronal ceroid lipofuscinosis gene *TPP1* and the hereditary motor and sensory neuropathy *DNMT1* gene, highlighting the genetic heterogeneity of spastic paraplegia. No plausible genetic cause was identified in 51% of probands, likely indicating the existence of as yet unidentified genes.

## Teaser

High-throughput next-generation sequencing can identify disease-causing mutations in extremely heterogeneous disorders. Kara *et al* . investigate a series of 97 index cases with complex hereditary spastic paraplegia (HSP). They identify *SPG11* defects in 30 families, as well as mutations in other HSP genes and genes associated with disorders including Parkinson’s disease.

## Graphical abstract

**Figure:**
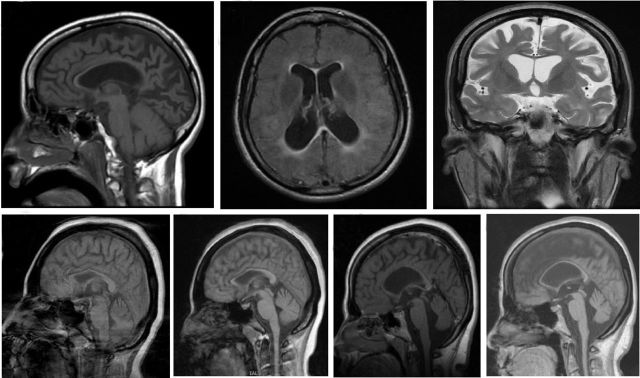
High-throughput next-generation sequencing can identify disease-causing mutations in extremely heterogeneous disorders. Kara *et al* . investigate a series of 97 index cases with complex hereditary spastic paraplegia (HSP). They identify *SPG11* defects in 30 families, as well as mutations in other HSP genes and genes associated with disorders including Parkinson’s disease.

## Introduction

The hereditary spastic paraplegias (HSPs) are a diverse group of neurodegenerative diseases with a prevalence of 2–7.4/100 000 in most populations ( Erichsen *et al.* , 2009 ; Blackstone, 2012 ; Noreau *et al.* , 2014 ). They can be inherited in autosomal dominant, autosomal recessive or X-linked patterns with an age of onset that varies from early childhood to 70 years of age. HSP was first classified by Harding in the early 1980s ( Harding, 1981 ), into pure or uncomplicated HSP, where lower limb spasticity occurs in isolation, frequently with bladder hyperactivity and mild impaired sense of vibration, and complex HSP that has prominent lower limb spasticity that is always accompanied by other neurological finding such as seizures, dementia, amyotrophy, ataxia, deafness, extrapyramidal disturbance, orthopaedic abnormalities and peripheral neuropathy ( Harding, 1981 ; Fink, 1993 , 2013 ; Blackstone *et al.* , 2011 ; Finsterer *et al.* , 2012 ).

Mutations in over 40 genes have been found to cause HSP ( de Bot *et al.* , 2010 , 2012 ; Dufke *et al.* , 2012 ; Coutinho *et al.* , 2013 ; Denora *et al.* , 2013 ; Loureiro *et al.* , 2013 ; Novarino *et al.* , 2014 ). The most common cause of autosomal dominant spastic paraplegia are *SPAST* /SPG4 mutations, with patients presenting with a pure form of HSP ( Schule and Schols, 2011 ; Finsterer *et al.* , 2012 ; Fink, 2014 ; Noreau *et al.* , 2014 ).

In the autosomal recessive complex HSP, the most frequent form seems to be associated with thinning of the corpus callosum ( Boukhris *et al.* , 2008 ; Finsterer *et al.* , 2012 ) and it is mostly due to mutations in *SPG11* ( Stevanin *et al.* , 2007 ; Paisan-Ruiz *et al.* , 2008 *a* , 2010 *b* ; Schule and Schols, 2011 ). It is also important to assess patients for rare causes of complex HSP such as enzyme deficiencies either biochemically or genetically ( Wu *et al.* , 2015 ). *SPG11* is clinically characterized by slowly progressive spastic paraplegia and cognitive decline usually beginning before the second decade of life. Four less common distinct phenotypes have also been associated with *SPG11* mutations, including Kjellin syndrome, which is a rare form of HSP with additional retinal manifestations ( Puech *et al.* , 2011 ; Nowak *et al.* , 2014 ), slowly progressive amyotrophic lateral sclerosis ( Orlacchio *et al.* , 2010 ; Daoud *et al.* , 2012 ), syndromes reminiscent of dystonia-parkinsonism ( Paisan-Ruiz *et al.* , 2010 *b* ; Kara *et al.* , 2013 ) and syndromes with prominent l -DOPA responsive parkinsonism ( Anheim *et al.* , 2009 *a* ; Everett *et al.* , 2012 *a* ). There have been reports of other types of spastic paraplegia being associated with improvement with l -DOPA ( Mallaret *et al.* , 2014 ). The increasing heterogeneity of spastic paraplegia and the clinical overlap seen with several other conditions suggests that there is still considerable genetic expansion to come in this group of disorders ( Beetz *et al.* , 2008 ; de Bot *et al.* , 2012 ).

The clinical heterogeneity of HSP reflects the contribution of diverse cellular pathways to their pathogenesis ( Crosby and Proukakis, 2002 ; Salinas *et al.* , 2008 ; Blackstone *et al.* , 2011 ). A number of proteins and pathways have been implicated including mitochondrial dysfunction (HSP60, spartin *,* paraplegin), microtubule trafficking and other membrane trafficking pathways (spastin, REEP1, atlsatin), lysosomal dysfunction (ZFYVE26), macroautophagy (spatacsin, ZFYVE26, AP5Z1) and lipid metabolism (FA2H, CYP7B1) ( Salinas *et al.* , 2008 ; Blackstone, 2012 ). The identification of macroautophagy is of particular interest, as autophagic dysfunction has been implicated in the pathogenesis of HSP ( Chang *et al.* , 2014 ), but also in a number of other neurodegenerative diseases such as Parkinson’s disease, Alzheimer’s disease, Huntington’s disease and the spinocerebellar ataxias ( Nixon, 2013 ). Given this mechanistic overlap and the presence of spasticity in other neurodegenerative and movement disorder phenotypes, defects in these genetic pathways are likely to overlap, particularly within processes involved in mitochondrial functions ( Schapira, 1999 ).

The aim of this study was 3-fold. First, to study the genotype–phenotype correlations and clinical features seen in a series of complex spastic paraplegia. We particularly focussed on *SPG11* , which makes up by far the largest group of complex spastic paraplegia cases. Second, to assess whether variants in genes that cause pure HSP, and other movement and neurodegenerative disorders are also involved in complex HSP. Third, following the identification of *SPG11* mutations as the most common cause of complicated spastic paraplegia, we investigated spatacsin (the protein product of *SPG11* ) through biochemical studies in a series of fibroblasts taken from patients and controls.

## Materials and methods

### Patients

A cohort of 97 patients that were referred to the National Hospital for Neurology and Neurosurgery (NHNN) for investigation or diagnosis were included in this study. Institutional review board (IRB)/ethical approval (UCLP – 99n102) and consent were obtained. We enrolled complex HSP patients and families where clinical details and DNA samples were available at the NHNN prior to 2015. From each family, we included only the proband. The inclusion criteria were slowly progressive HSP as the earliest manifestation or as the most significant clinical finding or the clinical reason for referral, along with at least one additional neurological feature such as: peripheral neuropathy, cognitive decline, epilepsy, skeletal/bony abnormalities, visual problems, parkinsonism, dystonia and ataxia ( Fink, 1993 , 2014 ). Nerve biopsy was carried out on one case and muscle biopsies on five cases ( Houlden *et al.* , 2001 ). Acquired or metabolic causes of HSP were excluded with an investigative work-up of MRI of the brain and spine, long chain fatty acids, white cell enzymes, routine and special blood tests for human T-lymphotropic virus (HTLV), Venereal Disease Research Laboratory test (VDRL), anti-nuclear antibodies (ANA)/extra nuclear antibodies (ENA)/anti-neutrophil cytoplasmic antibodies (ANCA), lupus and electromyography (EMG)/nerve conduction studies (NCS) and somatosensory evoked potentials (SSEP)/visual evoked potentials (VEP)/auditory evoked potentials (AEP) often early in the diagnosis. When referring to overall severity of clinical signs we used mild, moderate, and severe. An example of this classification is with urinary problems where mild signs would be untreated urgency or frequency symptoms, moderate as therapeutically treated symptoms, and severe when a long-term catheter of different types is required. The overall degree of disability severity was measured with the modified Rankin score where mild is <2.0, moderate is 2.5–3.5 and severe ≥4. This scale is used for measuring the degree of disability in the daily activities of people who have suffered any causes of neurological disability. The scale runs from 0–6, ranging from perfect health without symptoms to death ( Bonita and Beaglehole, 1988 ).

### Sanger sequencing

Sanger sequencing of the entire coding region of *SPG11* was carried out as previously described ( Stevanin *et al.* , 2007 ). Primer sequences and conditions are listed in Supplementary Tables 4 and Supplementary Data . When a mutation was identified in a familial case, DNA samples from available family members were also analysed by Sanger sequencing to assess segregation and to determine the phase in cases with compound heterozygous mutations ( Table 1 and Supplementary Table 1 ). *SPG11* mutations were named following the transcript NM_025137.3. For one case (Case 52) in which *SPG11* was negative for mutations, subsequent homozygosity mapping indicated *FA2H* as a candidate gene, which was found to be defective in this family. Multiplex ligation-dependent probe amplification (MLPA) was carried out using probes for *SPG11* [P306 kit (MRC Holland)] in 42 patients negative for mutations in *SPG11* . A sample was considered negative when all probes were within 0.75–1.25 copies and standard quality control criteria were met. Variants identified using next generation sequencing were also confirmed through Sanger sequencing.

**Table 1:** *SPG11* variants identified with clinical details

Proband number	Variant	Variant type	Ethnic origin	Consangunity	Family history	Age at onset	Current age	Gender	Other features
1	c.275_284del, p.R93Afs*25/c.6899T>C/p.L2300P	Compound heterozygous ^a^	UK	No	Yes	10	18	M	Early inturning of the left foot
2	c.2146C>T, p.Q716*	Homozygous	Pakistan	Yes	No	Child	26	F	Psoriasis
3	c.4132delA, p.S1378Afs*11/c.2843+1G>T	Compound heterozygous	UK	No	No	N/A	27	M	
4	c.7000G>C, p.A2334P/ c.3146-1G>C	Compound heterozygous ^a^	Italian/ Argentina	No	Yes	23	39	F	
5	c.3809T>A, p.V1270D	Homozygous ^a^	Turkish	Yes	Yes	12	18	M	Feet turn inwards, walk on tiptoes
6	c.5769delT, p.S1923Rfs*28	Homozygous ^a^	Kenya/ India/UK	Yes	Yes	20	33	F	Distant cousins also affected
7	c.5866+1G>A	Homozygous	Egyptian	Yes	No	4	35	F	Hand tremor
8	c.3623C>T, p.P1208L/c.852_856delCTTAA, p.N284Kfs*14	Compound heterozygous ^a^	UK	No	No	19	25	F	Elevated creatine kinase
9	c.6658_6659delAT, p.M2220Dfs*27	Homozygous ^a^	Cypriot	No	Yes	21	42	F	Brother SPG11 parkinsonism
10	c.782C>A, p.S261*	Homozygous	Pakistani	Yes	No	21	41	F	Factor VII deficiency, severe optic atrophy.
11	c.1492C>T, P.Q498*	Homozygous	Egyptian	Yes	Yes	18	20	F	Epilepsy
12	p.Q716*; p.Q845*	Compound heterozygous	Indian	Yes	Yes	Teen	26	F	
13	c.1235C>T, p.S412L	Homozygous	Egyptian	Yes	Yes	5	19	F	
14	c.1492C>T, p.Q498*	Homozygous	Egyptian	Yes	Yes	15	20	M	
15	c.3741dupA, p.P1248Tfs*17/ c.6091C>T, p.R2031*	Compound heterozygous ^a^	UK	No	Yes	5	24	M	Very slow to walk and talk
16	c.398delG, p.C133Lfs*22	Homozygous ^a^	Iranian	Yes	No	17	35	F	Severe pain
17	p.T206Nfs*13/p.W1524Lfs*22	Compound heterozygous	UK	No	No	14	23	M	Motor decline
18	p.M245Vfs*2/p.Y1238Lfs*27	Compound heterozygous	UK	No	No	Teen	39	M	
19	c.7115T>A, p.L2372*/ c.1471_1472delCT, p.L491Dfs*66	Compound heterozygous ^a^	UK	No	No	15	35	M	One episode encephalomyelitis
20	c.5769delT, p.S1923Rfs*28	Homozygous	Kenya/ India/UK	Yes	Yes	14	19	M	
21	c.315delC, p.A106Lfs*15	Homozygous ^a^	Iraqi	Yes	No	17	29	M	Bilateral cataracts
22	c.6891_6893delGAT, p.I2298del/ c.4237delinsTA, p.V1413Yfs*14	Compound heterozygous ^a^	UK	No	No	13	26	F	
23	c.2834+1G>T/ c.6754+3insTG	Compound heterozygous ^a^	UK	No	No	13	26	F	Baclofen pump
24	c.733_734delAT, p.M245Vfs*2	Homozygous	Pakistan	Yes	No	16	25	M	
25	c.1348dupA, p.I450Nfs*26/ c.5454_5455delAG, p.E1819Afs*10	Compound heterozygous ^a^	UK	No	Yes	10	50	M	
26	c.5399_5407delAGATinsTGGAGGAG, p.Q1800Lfs*31	Homozygous	Pakistan	Yes	Yes	13	33	F	Presented with cognitive problems
27	c.5623C>T, Q1875*/ c.7158dup, p.Q2387Tfs*6	Compound heterozygous	UK	No	No	27	33	F	Cerebellar tonsilar ectopia
28	c.267G>A, p.W89*	Homozygous	Pakistan	Yes	No	4	28	F	Reduced visual acuity and slow tongue movements
29	c.733_734delAT, p.M245V*2	Homozygous ^a^	India	Yes	Yes	12	25	F	
30	c.4483G>T, p.E1495*/c.5456_5457del, p.E1819Alafs*10	Compound heterozygous ^a^	UK	No	No	15	22	F	Generalized tonic clonic seizures
6 ^b^	c.5769delT, p.S1923Rfs*28	Homozygous ^a^	Kenya/India/UK	Yes	Yes	10	30	M	Oromandibular dystonia

* = nonsense; del = deletion; n/a = not available.

^a^ Other family members available for segregation.

^b^ Potentially related to patient number 6.

*SPG11* variants were labelled according to the transcript NM_025137.3 using the standard mutation nomenclature used in molecular diagnostics (Ogino *et al.* , 2007). See main text for discussion on pathogenicity.

### Next generation sequencing

A total of 66 patients were analysed that were either Sanger negative for *SPG11* mutations, carried a single heterozygous mutation or were more recently identified cases. These were analysed using either the Illumina next generation clinical exome (Trusight one) sequencing (Illumina Inc) targeting 4813 genes where target genetic regions were covered at least 30× in over 99% of the regions analysed, and seven patients underwent diagnostic Illumina whole exome sequencing, where coverage of the targeted genes was high though the Trusight clinical exome was superior. For data analysis, the raw data were mapped to the hg19 human reference assembly using the NovoAlign software, and polymerase chain reaction (PCR) duplicates were removed using the Picard software. Insertions-deletions (indels) and single nucleotide variants were called using the GATK package or SAMtools, and variants annotated using ANNOVAR, as previously described ( Hersheson *et al.* , 2013 ). In the preliminary filtering, variants with a minor allele frequency over 1/1000 in dbSNP ( http://www.ncbi.nlm.nih.gov/snp/ ) or in the ExAC database ( http://exac.broadinstitute.org/ ), synonymous variants and variants that were present in a segmental duplication region of over 95% were excluded. We focused on a subset of genes in which mutations have been previously associated with spastic paraplegia, neurodegeneration, ataxia, peripheral neuropathy, Parkinson’s disease and pallidopyramidal syndromes. Except in Case 48 where DNA was not available for Sanger, probable variants were confirmed through Sanger sequencing and were assessed for segregation in other affected or unaffected family members.

### Studies on patient-derived fibroblasts

Skin biopsies were obtained from eight affected patients with homozygous or compound heterozygous mutations in *SPG11* and nine healthy control subjects. Cases and controls were matched by gender, age and passage number (indicating the number of times a particular cell line has been subcultured and is used as a proxy for the age of the cells in culture) to the extent possible. Details of the cell lines used in this study are summarized in Supplementary Table 6 . Fibroblasts were grown as previously described ( Tucci *et al.* , 2014 ) and reverse transcriptase PCR was used to assess the transcription of *SPG11* in fibroblast cell lines ( Cottenie *et al.* , 2014 ). Primers spanning exons 7–8 of *SPG11* were designed to avoid non-specific amplification of genomic DNA. *GAPDH* was used as a housekeeping gene (see Supplementary Tables 4 and Supplementary Data for primer sequences and conditions). Autophagy was assessed through western blot analysis of autophagy and lysosomal markers including LAMP1, LC3, p62, HSP70 as previously described ( Manzoni *et al.* , 2013 ) ( Supplementary material ). LAMP1 is a structural component of lysosomes and consequently it can be used as a marker for lysosomal size and number. LC3-II is considered a marker for macroautophagy ( Tanida *et al.* , 2008 ). p62 is a cargo protein that binds to proteins targeted to autophagosomes for degradation ( Mizushima and Komatsu, 2011 ). The HSP70 family of proteins, in particular Hsc70, participate in chaperone-mediated autophagy promoting internalization of targeted proteins in lysosomes through LAMP2A ( Agarraberes *et al.* , 1997 ). Among the substrates of mTOR phosphorylation, we selected P70S6K as a marker of efficient starvation. The phosphorylated form of P70S6K decreases during starvation and can be used as a marker for the efficiency of the starvation experiments. P70S6K is a phosphorylation substrate only for mTOR ( Nixon, 2013 ) and is thus specific to check for mTOR block by starvation. Each experiment was repeated at least three times.

## Results

### Genetic findings

**Figure 1:**
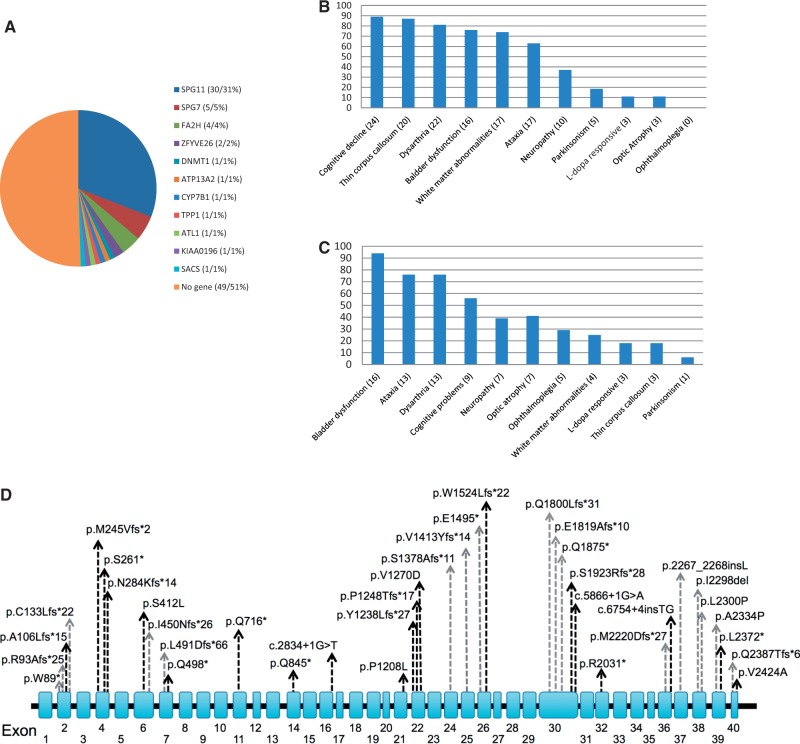
**Overview of mutations identified in spastic paraplegia genes.** ( **A** ) Pie chart showing the frequency of mutations in spastic paraplegia genes identified. The figures in brackets represent the number and percentage of cases respectively. ( **B** ) Frequencies of clinical features that were present in addition to the spastic paraplegia in SPG11 probands. ( **C** ) Frequencies of clinical features that were present in addition to spastic paraplegia in the spastic paraplegia patients with other mutations. In **B** and **C** the figures in brackets refer to the number of cases. ( **D** ) Diagram of the *SPG11* gene with mutations identified in the present study. Grey arrows indicate novel and black arrows indicate previously reported mutations.

A likely pathogenic genetic defect was identified in 48/97 (49%) of complex HSP patients ( Fig. 1 A, Table 1 and Supplementary Table 1 ). This does not include variants of unknown significance. Homozygous or compound heterozygous mutations in *SPG11* were identified in 30.9% of patients (30/97), which is the largest series to date and the most common cause of disease in complex HSP in the UK ( Fig. 1 D). No cases carrying copy number variants within the *SPG11* locus were identified using MLPA. The vast majority of *SPG11* mutations were non-sense or frameshift changes. Interestingly no homozygous mutations are present in the ExAC database ( http://exac.broadinstitute.org/gene ) of over 100 000 control population cases, indicating that loss of function mutations are not tolerated in the general population. *SPG11* was followed by *SPG7* (5/97), *FA2H* / *SPG35* (4/97), *ZFYVE26* / *SPG15* (2/97) and single families with *SPG3a* ( *ATL1* ), *SPG8* ( *KIAA0196* ), *SACS* and *SPG5* ( *CYP7B1* ) ( Fig. 1 A). Variants within genes associated with Parkinson’s disease (PARK9; *ATP13A2* ), neuronal ceroid lipofuscinosis (NCL; *TPP1* ) and hereditary neuropathy ( *DNMT1* ) were also identified. From the variants identified, nine in *SPG11* , and one in *FA2H* and one in *KIAA0196* had been previously reported ( Paisan-Ruiz *et al.* , 2008 *a* , *b* , 2010 *b* ; Dick *et al.* , 2010 ; Everett *et al.* , 2012 *a* ; Bettencourt *et al.* , 2013 ; Nowak *et al.* , 2014 ). Variants in other non- *SPG11* genes were not found in the ExAC population control database at a frequency of <10 heterozygous cases, except for Cases 38, 39, 44 and 48. Case 48 carries two *CYP7B1* likely compound heterozygous variants, where one (p.R486C) is reported as pathogenic ( Goizet *et al.* , 2009 ) and present in 82 ExAC population individuals and the other variant (c.122+2T>C) is absent in this dataset. Cases 38, 39 and 44 carry the SPG7 variant p.A510V, which was present in 57 individuals on the ExAC database but has previously been multiply-reported as pathogenic ( Choquet *et al.* , 2015 ; Pfeffer *et al.* , 2015 ). In addition, the SPG7 variants we identified and those previously reported, often had a similar phenotype of spastic ataxia but we also highlight the prominent opthalmoplegia ( Choquet *et al.* , 2015 ). Variants of unknown significance were also identified ( Supplementary Table 3 ) and are discussed in detail in the Supplemental material and a summary of the negative case details is given in Supplementary Fig. 3 .

### Clinical findings

In the 97 individuals identified with complex HSP, the clinical phenotypes primarily consisted of HSP with ataxia as the most common association, followed by cognitive decline, neuropathy, seizures, dystonia, parkinsonism and rarely other features. The mean age of onset was 16 years, ranging from age 3 to 39 years. Summary clinical information for the complex HSP cases is given in Table 1 and Supplementary Tables 1–3 .

### SPG11

#### General features

*SPG11* mutations were identified in 30 probands. We removed one proband that may be distantly related to Family 6 because they come from the same ethnic community group and have the same mutation. In general, *SPG11* onset was in childhood/early teenage years (mean age at onset 14.3 years, range 4–27 years), with walking problems and spasticity, severe bladder problems, ataxia, neuropathy, parkinsonism and/or cognitive problems ( Table 1 and Fig. 1 B).

#### Atypical presentation

Some patients presented in an atypical way, such as the proband from Family 5 ( Table 1 ) who exhibited a very mild phenotype including toe walking, brisk reflexes and extensor plantars at the age of 12 years with little disease progression by the age of 22 years. His aunt had typical *SPG11* features in her 30s. Case 10 had spastic paraplegia with severe optic atrophy and visual problems. The proband from Family 6 had a severe and treatment-resistant oromandibular dystonia. Interestingly his sister had a relatively mild phenotype but his distant community cousins had a typical severe *SPG11* . The longest surviving case carrying an *SPG11* mutation is currently 50 years of age and has severe complex HSP as well as severe cognitive decline and late-onset seizures, similar to the deceased sibling (Family 25).

#### Genotype–phenotype correlations

There were no clear genotype–phenotype correlations that we could define although Case 5 with the mildest clinical and MRI phenotype had a homozygous missense mutation, and progressed relatively slowly.

#### MRI findings

**Figure 2:**
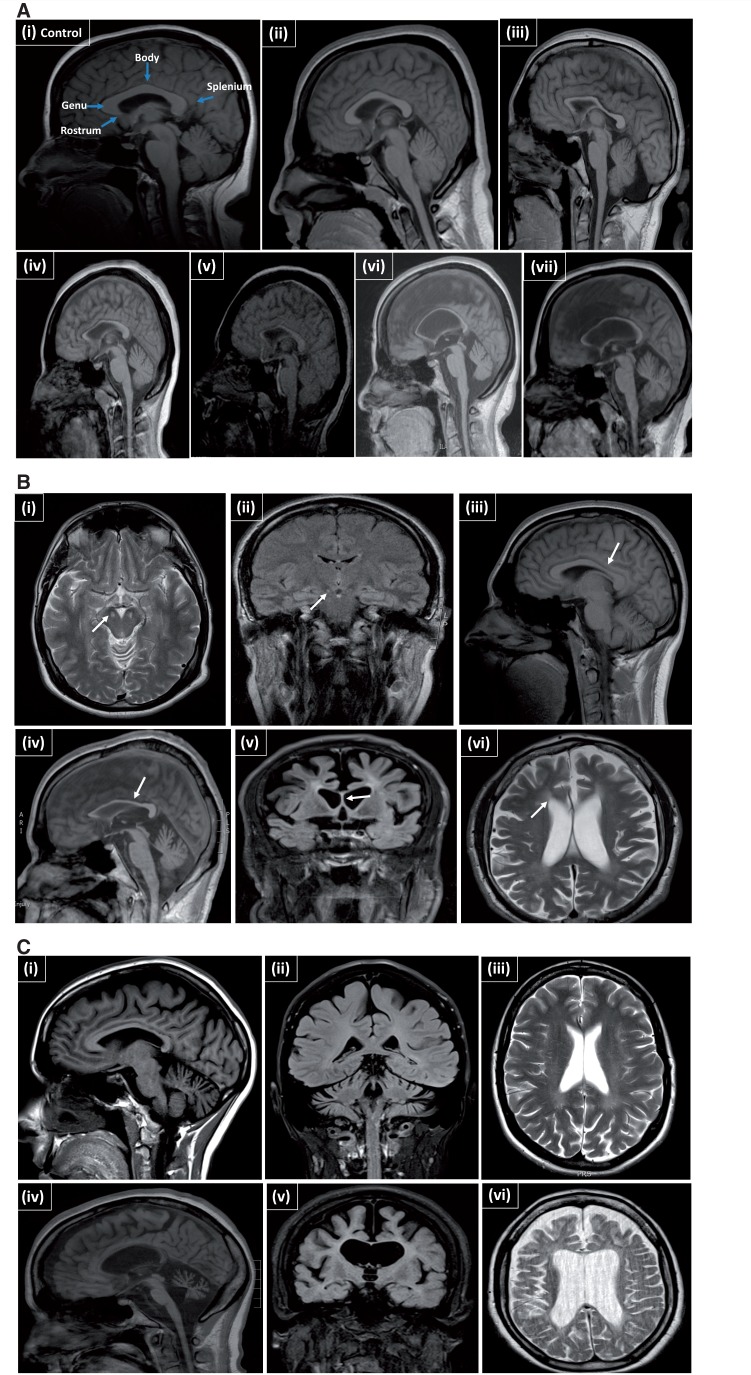
**MRI features in patients with complex HSP.** ( **A** ) Sagittal MRI of SPG11 patients showing progressive thinning of the corpus callosum and cerebral atrophy, which correlates with the progression of clinical features. ( **i** ) MRI from a healthy individual with labelling of the different parts of the corpus callosum. ( **ii** ) Case 5 at age 18 (mild disease). ( **iii** ) Case 8 at age 22 (mild-moderate disease). ( **iv** ) Case 17 at age 23 (moderate disease). ( **v** ) Case 10 at age 30 (severe disease). ( **vi** ) Case 16 age 32 (severe disease). ( **vii** ) Case 9 age 39 (severe disease). See Table 1 for details of case numbers. ( **B** ) MRI of complex HSP cases. ( **i** ) Case 37 (age 24 years), SPG7 showing an axial MRI with high signal in the cerebral peduncles (arrow) and on coronal imaging ( **ii** ) and sagittal imaging (arrow) ( **iii** ) with thinning of the body of the corpus callosum (arrow). Case 33 (age 34 years) ( **iv** to **vi** ), SPG15 with thinning of the corpus callosum ( **iv** ) (arrow) and generalized atrophy with periventicular white matter abnormalities (arrow). ( **C** ) Sagittal MRI of *FA2H* patients. ( **i–iii** ) Case 32, age 32 years, slowly progressive with thinning of the corpus callosum, cerebellar and cortical atrophy. ( **iv–vi** ) Case 52, age 37 years, more rapid and severely affected: shows severe corpus callosum thinning, cerebellar and cerebral atrophy, but preserved white matter, similar to the Case 32.

The MRI findings in *SPG11* patients are presented in Fig. 2 A; images were compared to controls. Patients with mild-to-moderate disease had minimal corpus callosum thinning while severe *SPG11* patients had more severe thinning as well as cerebral atrophy. When the disease is severe the corpus callosum remains at a static state and does not seem to change over time, as in Supplementary Fig. 1 , although these are longitudinal data from only two cases.

### SPG7

#### General features

Five families were identified with *SPG7* variants (Families 35, 37–39 and 44). In the Kenyan family (Family 35), presentation was in the 30s with progressive spasticity and ataxia. In the other families, disease onset was earlier, often with an initial diagnosis of cerebral palsy or poor coordination. Associations of *SPG7* variants with optic atrophy, neuropathy and ophthalmoplegia were also identified.

### *SPG35* ( *FA2H* )

#### General features

Four families with *FA2H* ( *SPG35* ) variants were identified (Families 32, 34, 51 and 52; Supplementary Table 1 ), of which three carried compound heterozygous variants and one a homozygous variant. In Families 32, 51 and 52 segregation was shown in affected and unaffected members. The age of onset varied between 5 and 22 years with the initial presentation being progressive balance problems and toe walking. Spastic quadriplegia was present on examination, along with early bladder problems, dysarthria, dysphagia and limb ataxia. One family had severe ophthalmoplegia and skew defect, seizures were present in two families, and two families had optic atrophy. Interestingly, the dysarthria progressed rapidly in all cases, developing into an early anarthria which is different than *SPG11* and *15* and an important clinical point to note.

#### MRI findings

MRI findings ranged from atrophy of the cerebellum and brain stem to more significant findings that also included corpus callosum atrophy, cortical atrophy and white matter abnormalities ( Fig. 2 C).

### *ZFYVE26* / *SPG15*

#### General features

Two cases with homozygous mutations in *ZFYVE26* / *SPG15* were identified. These cases presented and progressed in a very similar way to *SPG11* , although the neuropathy was greater ( Supplementary Table 1 and Figs 2 B and 4 ). In the non- *SPG11* cases there were also additional neurological features, as shown in Fig. 1 C ***.***

**Figure 3:**
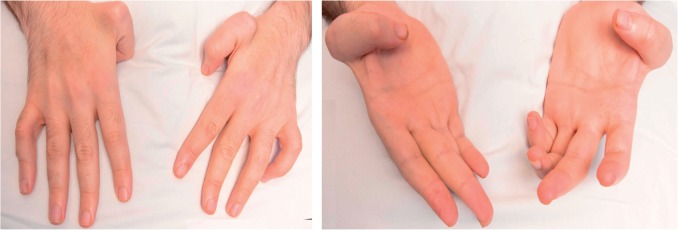
Photographs of the hands of Case 33 with *ZFYVE26/SPG15* mutation (homozygous p.R1378* mutation). Showing the adducted thumbs that are similar to those seen in MASA (mental retardation, aphasia, shuffling gait and adducted thumbs) syndrome. Age of patient: 34 years.

**Figure 4:**
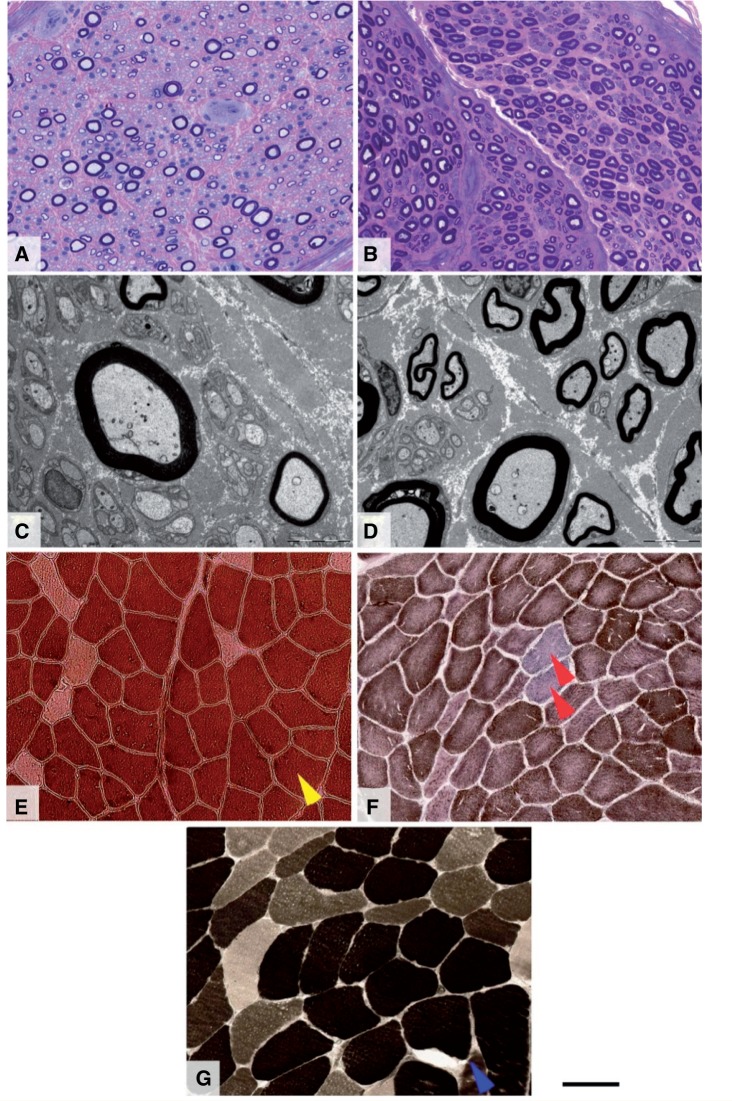
**Morphological findings of one nerve and four muscle biopsies in five genetically characterized patients with HSP.** Patient with *ZFYVE26/SPG15* mutation ( **A** and **C** ) and control individual ( **B** and **D** ). *SPG11* mutation ( **E** ), *SPG7* mutation ( **F** and **G** ). Semi-thin resin preparations stained with methylene blue azure—basic fuchsin ( **A** and **B** ) show a reduction of large myelinated fibre density in the patient’s biopsy, compared with a biopsy from age-matched control. Large fibre loss is further confirmed by electron microscopy ( **C** and **D** ), while unmyelinated fibres are better preserved. There is no evidence of active axonal degeneration, regeneration or demyelination and the overall picture is that of chronic axonal neuropathy. The muscle biopsies from three patients investigated for signs of denervation show varied appearances. In the biopsy from the patient with a known *SPG11* mutation ( **E** ) there is predominance of type 1 fibres (yellow arrow, ATP pH4.3). In one patient with *SPG7* mutation ( **F** ) the most significant finding in the muscle biopsy is that of several COX-deficient fibres (red arrows) seen on combined COX-SDH preparation. In another patient with *SPG7* mutation ( **G** ) the biopsy confirms neurogenic change with evidence of previous denervation with re-innervation (blue arrow indicates a group of type 1 fibres, ATP pH4.3). Scale bars: **A** , **B** and **E–H** = 40 μm; **C** and **D** = 5 μm.

#### Atypical presentation

In addition, Case 33 has adducted thumbs that were very similar to those seen in mental retardation, aphasia, shuffling gait, and adducted thumbs (MASA) syndrome ( *SPG1* ), an X-linked spastic paraplegia syndrome that is caused by mutations in the *LICAM* gene ( Jouet *et al.* , 1994 ; Vits *et al.* , 1994 ) ( Fig. 3 ).

### ATP13A2

#### General features

The proband from Family 41 presented at 18 years old with spastic quadraplegia, falls, cognitive decline, bilateral pes cavus and ataxia. Eye signs were prominent, with bilateral divergent squints and nystagmus on lateral gaze and reduced upgaze. He was the product of a consanguineous marriage. Genetic testing revealed novel heterozygous variants in *ATP13A2* (c.3017_3019del; p.1006_1007del), which are near/in the transmembrane helix and not present in over 100 000 ExAC controls.

#### MRI findings

The MRI showed cerebral atrophy and subtle abnormalities of the basal ganglia. An l -DOPA trial initially helped patient symptoms but there were no signs of objective or long-term improvement.

### TPP1

#### General features

In Family 43, which was found to carry a variant in *TPP1* , the proband initially presented with walking problems, progressive spastic paraplegia and poor intellectual function at the age of 11 years old, with a past history of two seizures. At the age of 31 she had severe spasticity in her limbs, along with a bulbar palsy, dystonic neck posturing and also severe cognitive problems. There is no family history.

#### MRI and additional tests

Her MRI showed cerebral atrophy and thinning of the corpus callosum ( Supplementary Fig. 1 ). There were background EEG abnormalities but the muscle and skin biopsy was normal. She responded to l -DOPA for 5 years that improved her mobility temporarily, though screening for variants in *GCH1* was negative.

### Nerve conduction studies, EMG and nerve biopsy

Nerve conduction studies and EMG were minimally helpful in defining some types of HSP ( Supplementary Table 2 ). One *SPG11* case was abnormal along with both *ZFYVE26* / *SPG15* cases. Similarly, a nerve biopsy was also abnormal in *ZFYVE26* / *SPG15* [Case 33, Fig. 2 B(iv–vi)].

### Characterization of the autophagic response in *SPG11* patient-derived fibroblasts

**Figure 5:**
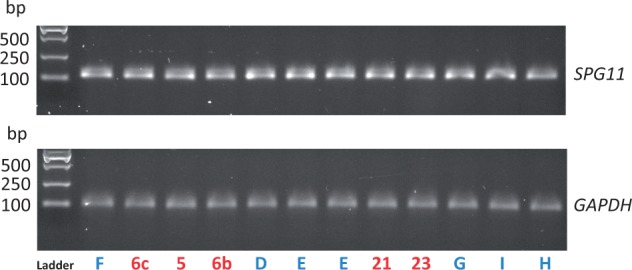
**
Reverse transcription PCR to assess *SPG11* mRNA expression in fibroblasts.** Wide expression is seen across SPG11 affected and control fibroblasts. Numbers in red are SPG11 affected cases from Table 1 and Supplementary Table 1 . Letters in blue are controls from Supplementary Table 1 . *GAPDH* = housekeeping gene glyceraldehyde 3-phosphate dehydrogenase.

**Figure 6:**
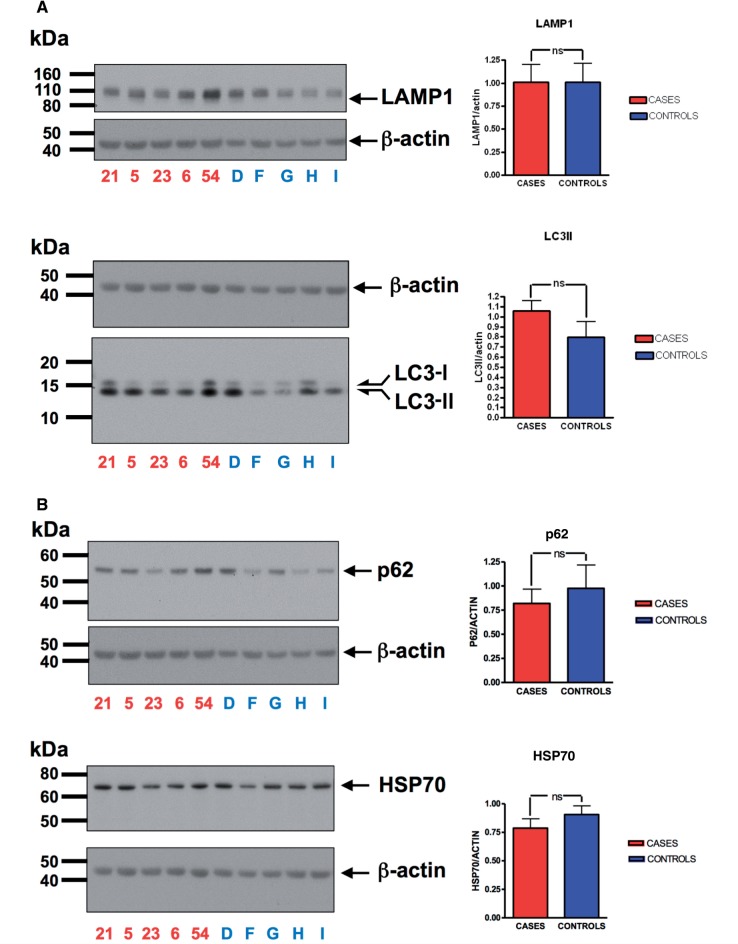
**Analysis of markers for autophagy and lysosomal function in human fibroblast cells.** ( **A** ) Fibroblast protein expression levels of LAMP1 and LC3 as compared to beta actin in *SPG11* affected and control fibroblasts after starvation induced autophagy (overnight serum starvation, followed by 2.5 h amino acid starvation in low glucose). ( **B** ) Fibroblast protein expression levels of p62 and HSP70 as compared to beta actin in *SPG11* affected and control fibroblasts after starvation induced autophagy (overnight serum starvation, followed by 2.5 h amino acid starvation in low glucose).

Reverse transcription PCR on RNA extracted from fibroblast cell lines from cases and controls showed that *SPG11* is expressed in this tissue at levels similar to the housekeeping gene *GAPDH* ( Fig. 5 ). We studied the different autophagy and lysosomal markers in fibroblasts under basal conditions ( Supplementary Figs 4–7 ) and after induction of autophagy by starvation ( Fig. 6 A and B, and Supplementary Figs 4–7 ). We did not observe significant correlations between disease status and autophagic/lysosomal markers, although there was a trend towards increased LC3-II in cases.

## Discussion

Here we describe the genetic analysis of 97 complex HSP probands using a combination of Sanger and next generation sequencing. We identified the genetic cause of the disease in 49% of the patients studied. *SPG11* defects were found to be by far the commonest cause of complex HSP in the UK, accounting for 30.9% of cases and being the largest series reported to date. This is higher than previous reports ( Stevanin *et al.* , 2007 , 2008 ; Erichsen *et al.* , 2008 ; Paisan-Ruiz *et al.* , 2008 *a* ; Anheim *et al.* , 2009 *a* ; Orlen *et al.* , 2009 ; Schule *et al.* , 2009 ; Orlacchio *et al.* , 2010 ; Southgate *et al.* , 2010 ; Guidubaldi *et al.* , 2011 ; Daoud *et al.* , 2012 ; Cao *et al.* , 2013 ), but the frequency is in-line with a recent study from Italy (26.2%) ( Pensato *et al.* , 2014 ). Variants in *SPG7* represented 6% and the frequency of *FA2H* was also higher that previous reports at 4% ( Pensato *et al.* 2014 ). *ZFYVE26* / *SPG15* was infrequent in our cohort with only two families although Family 33 extends the clinical features associated with variants in this gene ( Figs 3 and 4 ). The discrepancy in the variants frequency is likely population-specific and possibly due to the use of next generation sequencing in our report, in contrast to previous candidate gene-based studies. In addition, our cohort was a multi-ethnic population, whereas those in previous reports were ethnically more homogeneous, focusing on the Mediterranean basin or Caucasian populations.

The core clinical presentation of *SPG11* was consistent with previous reports, but a number of other neurological features were identified ( Fig. 1 B). The age of onset ranged between 4 and 27 years, most frequently presenting with walking problems due to lower limb spasticity and later ataxia and foot deformities. Other progressive neurological features include parkinsonism that often responded to l -DOPA, axonal neuropathy and learning difficulties ( Fig. 1 B). In addition *SPG11* cases tended to remain mobile up until 30 years, with a mean modified Rankin score of <3. Patients aged over 30 years were more significantly disabled and completely dependent when over 40 years ( Supplementary Fig. 2 ) ( Patel *et al.* , 2012 ).

One *SPG11* patient with extreme visual problems was found to have Kjellin syndrome, although optic atrophy and retinal signs were generally rare in this HSP *(*Puech *et al.* , 2011 ; Nowak *et al.* , 2014 ). The majority of patients had white matter abnormalities and corpus callosum thinning on MRI, though this feature was not restricted to *SPG11* mutations as we also observed it in *ZFYVE26* / *SPG15* and *FA2H* variant carriers ( Fig. 2 A–C). Although in a relatively low number of patients, *SPG11* was associated with parkinsonism in five cases and was l -DOPA-responsive in three cases, indicating a role for the basal ganglia in HSP, suggesting that this medication should be considered in *SPG11* -associated HSP. Two patients had a positive family history of essential tremor (Case 6) and Parkinson’s disease (Case 15), an observation that has previously been made in other families ( Kang *et al.* , 2004 ; Anheim *et al.* , 2009 *b* ; Paisan-Ruiz *et al.* , 2010 *a* ; Guidubaldi *et al.* , 2011 ; Everett *et al.* , 2012 *b* ). Other clinical features that we found in patients with *SPG11* mutations include telangiectasia (Case 9) and bilateral cataracts (Case 21). We did not observe a predilection for males in comparison to females; thus, gender-specific factors are not likely to contribute to the pathogenesis of *SPG11* mutations, contrary to other genetic forms of HSP ( Proukakis *et al.* , 2011 ).

A number of the mutations identified within *SPG11* were previously undescribed. The majority of these mutations were nonsense, distributed throughout the coding length of the gene, supporting a loss-of-function mechanism underlying disease. One family with mild disease was found to carry a previously reported homozygous missense mutation p.V1270D (Case 5) ( Conceicao Pereira *et al.* , 2012 ) altering a highly conserved residue and segregating with disease, and this may suggest that missense mutations lead to a milder phenotype although certainly more cases are required to prove this. No patients carried larger genomic rearrangements and similarly, previous studies have only very rarely identified such mutations within *SPG11* ( Bauer *et al.* , 2009 ; Crimella *et al.* , 2009 ; Denora *et al.* , 2009 ; Conceicao Pereira *et al.* , 2012 ).

In our cohort, *FA2H* variants were associated with a spectrum of disability ranging from mild through to severely affected. In all four families, affected members exhibited dysarthria that rapidly progressed to anarthria, which is a feature that could be used as a diagnostic clue to initiate genetic testing for *FA2H* variants. Interestingly, *FA2H* variants have been previously associated with neurodegeneration with brain iron accumulation ( Kruer *et al.* , 1993 , 2010 ). It is unknown what factors could influence the development of iron accumulation in the brain in association with this variant. A number of unusual HSP associations were identified with variants in genes such as *DNMT1* , *ATP13A2* and *TPP1* , which warrants wider genetic testing of complex HSP, with *SPG11* being the first candidate, followed by the other three genes ( *FA2H* , *SPG7* , *ZFYVE26* / *SPG15* ).

The patient with the *ATP13A2* variant had a progressive HSP with ataxia and cognitive problems but no parkinsonian features seen and the patient with *TPP1* variants had a complex HSP that was considered likely to be *SPG11* . In this case *SPG11* was Sanger sequenced several times and deletion analysis carried out until the *TPP1* variant was identified. Variants in *TPP1* and *ATP13A2* have been previously identified in patients with NCL although not with a HSP phenotype ( Schnabel *et al.* , 1991 ; Sleat *et al.* , 1997 ; Bras *et al.* , 2012 ). *ATP13A2* variants are associated with heterogeneous phenotypes including Kufor-Rakeb syndrome ( Ramirez *et al.* , 2006 ), NCL ( Bras *et al.* , 2012 ) and Parkinson’s disease ( Malakouti-Nejad *et al.* , 2014 ). Kufor-Rakeb syndrome clinically partially overlaps with HSP as patients often exhibit spasticity in addition to dystonia, parkinsonism and mental retardation ( Ramirez *et al.* , 2006 ). Patients with NCL caused by *ATP13A2* variants have similar clinical features with Kufor-Rakeb syndrome patients ( Bras *et al.* , 2012 ). NCL syndromes are characterized by accumulation of autofluorescent material, a feature that is not characteristic of HSP, though biopsy material was not as yet available for our HSP patient with the *ATP13A2* variant. In general, these findings suggest an overlap between spastic paraplegias and numerous other neurodegenerative diseases, an observation that has already been made through protein network analyses ( Novarino *et al.* , 2014 ). Our results also highlight the worldwide distribution of mutations in genes associated with autosomal recessive spastic paraplegias and these data indicate that ethnic background should not be a criterion to prioritize patients for genetic testing. These findings expand the clinical spectrum of complex spastic paraplegias and underline the clinical overlap with other diseases such as spinocerebellar ataxias and dystonia-parkinsonism and the difficulty of prioritizing genetic testing in these patients where functional investigation should ideally be applied to all likely pathogenic variants. Thus, the use of next generation sequencing-based panels containing a series of candidate genes is becoming exceedingly important in the genetic work-up of such patients.

Numerous variants of unknown significance were detected, which is a common problem associated with the application of high throughput approaches to study the genetic basis of diseases ( MacArthur *et al.* , 2014 ). These cases could represent a broadening of the clinical phenotype but future studies are necessary to clarify this. We did not identify definite or probable variants in any of the genes studied in 51% of the cases. This observation indicates that there are probably additional rare variants in novel genes causing spastic paraplegia that have not yet been discovered. Alternatively, it is possible that other types of genetic mechanisms such as mosaicism ( Proukakis *et al.* , 2013 ), di- or polygenic inheritance and imprinting defects could be responsible for the disease in a proportion of patients.

The functional role of spatacsin is unknown, though candidate gene pathway analysis has implicated autophagic dysfunction in the pathogenesis of the disease ( Chang *et al.* , 2014 ). We attempted to replicate previous findings linking spatacsin with the regulation of autophagy using a cohort of patient-derived fibroblasts. Although a trend towards an increase in LC3-II was present in cases, no significant changes in autophagy markers of disease were identified in these cells under the experimental conditions used herein to assess autophagy. It should be noted, however, that interpretation of results from functional studies on patient fibroblasts requires caution, as interindividual variability is an important limitation that could potentially mask real effects of mutations or, alternatively, lead to false positives. Further studies with an increased number of cases and controls are therefore required to determine whether spatacsin has a role in the regulation of autophagy, including detailed analysis of autophagic flux following treatment with bafilomycin *.* Such functional characterization of spatacsin will be crucial to understanding the mechanisms underlying degeneration in these cases, as well as developing potential therapies. While outside the scope of this present study, this should be a priority for the research community in the future.

## Supplementary Material

Supplementary Data

## Abbreviations

HSP: hereditary spastic paraplegia
NCL: neuronal ceroid lipofuscinosis
