# Supplementary material for: Genetic and phenotypic characterization of complex hereditary spastic paraplegia
Source: Brain. 2016 May 23;139(7):1904–18. doi: 10.1093/brain/aww111 (PMC4939695; doi:10.1093/brain/aww111)
Supplement: Supplementary Data [file aww111_supplementary_data.zip › brain-2015-01890-File007.pdf]

Table S2	Motor nerve conduction studies									Sensory nerve conduction studies						EMG		Summary findings of NCS and EMG
	Median nerve			Ulnar nerve			Tibial nerve			Median nerve		Ulnar nerve		Sural nerve		Limbs. Upper:lower		
Proband number	Distal latency (msec)	CMAP amplitude (uV)	MCV (m/s)	Distal latency (msec)	CMAP amplitude (uV)	MCV (m/s)	Distal latency (msec)	CMAP amplitude (uV)	MCV (m/s)	SNAP amplitude (uV)	SCV (m/s)	SNAP amplitude (uV)	SCV (m/s)	SNAP amplitude (uV)	SCV (m/s)	Chronic denervation		
**SPG 11 patients**																		
**2**	3.3	10.7	55	2.4	11.9	58	4	3.9	53	25	62.5	23	59	14	41.5	No	No	Normal NCS/EMG
**6**	3.2	8.3	56	2.3	8.9	62	2.8	3.7	45	23	65	15	64	12	48	N/A	No	Normal NCS/EMG
**5**	4	4.9	51	2.4	10	61	3.4	5.2	48	28	61	13	67	11	55	No	No	Normal NCS/EMG
**8**	3.9	7.3	64	N/A	N/A	N/A	4.8	5.3	45	19	61	9	50	6	55	No	Mild	Mild neuropathy
**10**	N/A	N/A	N/A	N/A	N/A	N/A	5.6	0.2	33	12	53	21	59	N/A	N/A	N/A	N/A	Normal NCS/EMG
**16**	4.1	6.6	52	N/A	N/A	N/A	5	3.4	42.5	15	57.5	14	53.5	14	41.5	No	No	Normal NCS/EMG
**21**	N/A	N/A	N/A	N/A	N/A	N/A	3.4	45	11.6	N/A	N/A	N/A	N/A	20	55	No	No	Normal NCS/EMG
**26**	3.6	6.1	53	3.8	3.8	43	5.9	3.3	35	28	54	19	56	9	40	Mod	Mod	Axonal neuropathy
**SPG7 patients**																		
**37**	3.2	9.1	N/A	N/A	N/A	N/A	4.2	2.9	43	32	57	16	63	12	52	N/A	N/A	Normal NCS/EMG
**39**	5.1	4.6	48	N/A	N/A	N/A	N/A	N/A	N/A	1	30	6	51	N/A	N/A	N/A	N/A	Mod Carpal tunnel
**ATP13A2**																		
**41**	4	9.5	58	2.7	12.4	N/A	5.8	1	40	15	56.5	12	55	32	40	No	No	Mod motor neuropathy
**FA2H**																		
**32**	N/A	N/A	N/A	N/A	N/A	N/A	3.6	15	48	N/A	N/A	N/A	N/A	31	58	No	No	Normal EMG/NCS
**34**	N/A	N/A	N/A	N/A	N/A	N/A	4.2	4.4	55	N/A	N/A	N/A	N/A	55	50	No	No	Normal EMG/NCS
**KIAA0196**																		
**45**	2.7	8.6	59	N/A	N/A	N/A	3.5	15.2	44	16	64	11	60	15	57	No	No	Normal EMG/NCS
**CYP7B1**																		
**48**	4.2	10.6	51	3.3	8.5	53	6.6	3.3	40	10	46	4	45	10	34	No	No	Normal EMG/NCS
**ATL1**																		
**31**	2.9	6.7	58	2.6	10.2	N/A	3.8	4.4	53	20	63	10	64	18	51	No	No	Normal EMG/NCS
**DNMT1**																		
**47**	3.1	12.4	59	N/A	N/A	N/A	4.3	4.5	52	39	55	15	55	23	47	N/A	N/A	Normal NCS/EMG
**ZFYVE26**																		

**33**	4	2.5	57	2.9	5.8	63	4.1	0.8	44	12	52	6	55	4	47	Yes	Yes	Motor neuropathy

Table S2: Nerve conduction studies (NCS) and electromyography (EMG) in HSP cases. Studies were all carried out at the same institution. Key: Compound Muscle Action Potential = CMAP), mod = moderate, microvolts (uV), metres per second = m/s, milliseconds = msec. See tables 1 and S1 and figures for the clinical details of the probands.
