# Supplementary material for: Genetic and phenotypic characterization of complex hereditary spastic paraplegia
Source: Brain. 2016 May 23;139(7):1904–18. doi: 10.1093/brain/aww111 (PMC4939695; doi:10.1093/brain/aww111)
Supplement: Supplementary Data [file aww111_supplementary_data.zip › brain-2015-01890-File006.pdf]

Case	Gene	Variant	Transcript	Variant type	Ethnicity	Consanguinity	Family history	Age at onset	Age at exam	Gender	Other
31	*ATL1*	c.1243C>T, p.R415W	NM_015915.4	Heterozygous	UK	No	No	Child	37	M	Broken pursuit and lobar cerebellar agenesis
32	*FA2H*	c.620C>T, p.T207M; c.460C>T, p.R154C	NM_024306.4	Compound heterozygous#	Italy/UK	No	no	22	31	F	Thin corpus callosum and atrophy
33	*ZFYVE26*	c.4132C>T, p.R1378*	NM_015346.3	Homozygous#	Pakistan	Yes	no	19	34	M	Thin corpus callosumand white matter abnormalities
34	*FA2H*	c.1051A>C, p.S351R; c.486G>C, p.E162D	NM_024306.4	Compound heterozygous	UK	No	No	3	18	F	General atrophy and white matter abnormalities
35	*SPG7*	c.233T>A, p.L78*	NM_003119.3	Homozygous	Kenya	Yes	Yes	39	62	M	
37	*SPG7*	c.1672A>T, p.K558*	NM_003119.3	Homozygous	UK	No	Yes	14	25	M	Asperger's and ADHD
38	*SPG7*	c.1523G>A, p.R508H / c.1529C>T, p.A510V	NM_003119.3	Compound heterozygous	UK	No	No	38	45	M	Slow saccades
39	*SPG7*	c.1450_1457del, p.E484Gfs*4/	NM_003119.3	Compound heterozygous	UK	No	No	46	70	F	
41	*ATP13A2*	c.3020_3022del, p.F1007del	NM_001141974.2	Homozygous	Pakistan	Yes	No	18	46	M	
43	*TPP1*	c.1525C>T, p.Q509*; c.1340G>A, p.R447H	NM_000391.3	Compound heterozygous	Wales	No	No	4	27	F	
44	*SPG7*	c.1529C>t; 2102A>C. p.A510V; p.H701P	NM_003119.3	Compound heterozygous	UK	No	Yes	16	66	F	
47	*DNMT1*	c.2053G>A, p.A685T	NM_001130823.1	Heterozygous	UK	No	Yes	22	43	F	
48	*CYP7B1*	c.1456C>T, p.R486C/ c.122+2T>C	NM_004820.3	Compound heterozygous	UK	No	No	25	62	M	Seizures and late onset migraine
50	*SACS*	c.5557_5561del, p.R1855Cfs*25	NM_001278055.1	Homozygous#	Kuwait	Yes	Yes	Child	23	M	
51	*FA2H*	c.798C>G, p.D266E; c.782dupA:p.H261Qfs*	NM_024306.4	Compound heterozygous#	Ireland/UK	No	Yes	4	25	M	Initially diagnosed as cerebral palsy. Later seizures
52	*FA2H*	c.157_174del, p.Arg53_Ile58del	NM_024306.4	Homozygous#	Pakistan	Yes	Yes	6	38	F	Thin corpus callosum, atrophy, white matter abnormalities
53	*ZFYVE26*	c.C4312T:p.R1438X	NM_015346.3	Homozygous	Ireland	No	Yes	16	26	M	
54	*KIAA0196*	c.677T>C, p.I226T	NM_014846	Heterozygous#	UK	No	Yes	10	28	F	

Supplementary table S1: Likely pathogenic variants identified in other genes apart from *SPG11* in our complex HSP series. Variants were labelled according to the gene trascript given and using standard mutation nomenclature used in molecular diagnostics (Ogino S et al., 2007). See main text for discussion on pathogenicity. Key: * = nonsense, del = deletion, n/a = not avaliable, # = other family members avaliable for segregation, ADHD = attention deficit hyperactivity disorder.
