# Supplementary material for: Genetic and phenotypic characterization of complex hereditary spastic paraplegia
Source: Brain. 2016 May 23;139(7):1904–18. doi: 10.1093/brain/aww111 (PMC4939695; doi:10.1093/brain/aww111)
Supplement: Supplementary Data [file aww111_supplementary_data.zip › brain-2015-01890-File005.pdf]

Supplementary figures S4-S7

Figures S4-S7: Western blots for LAMP1, LC3I, LC3II, p63 and HSP70 where starved and fed conditions are depicted on the same gel. Induction of autophagy was achieved by removal of amino acids and serum for 2.5 hours in low glucose conditions.

Figure S4

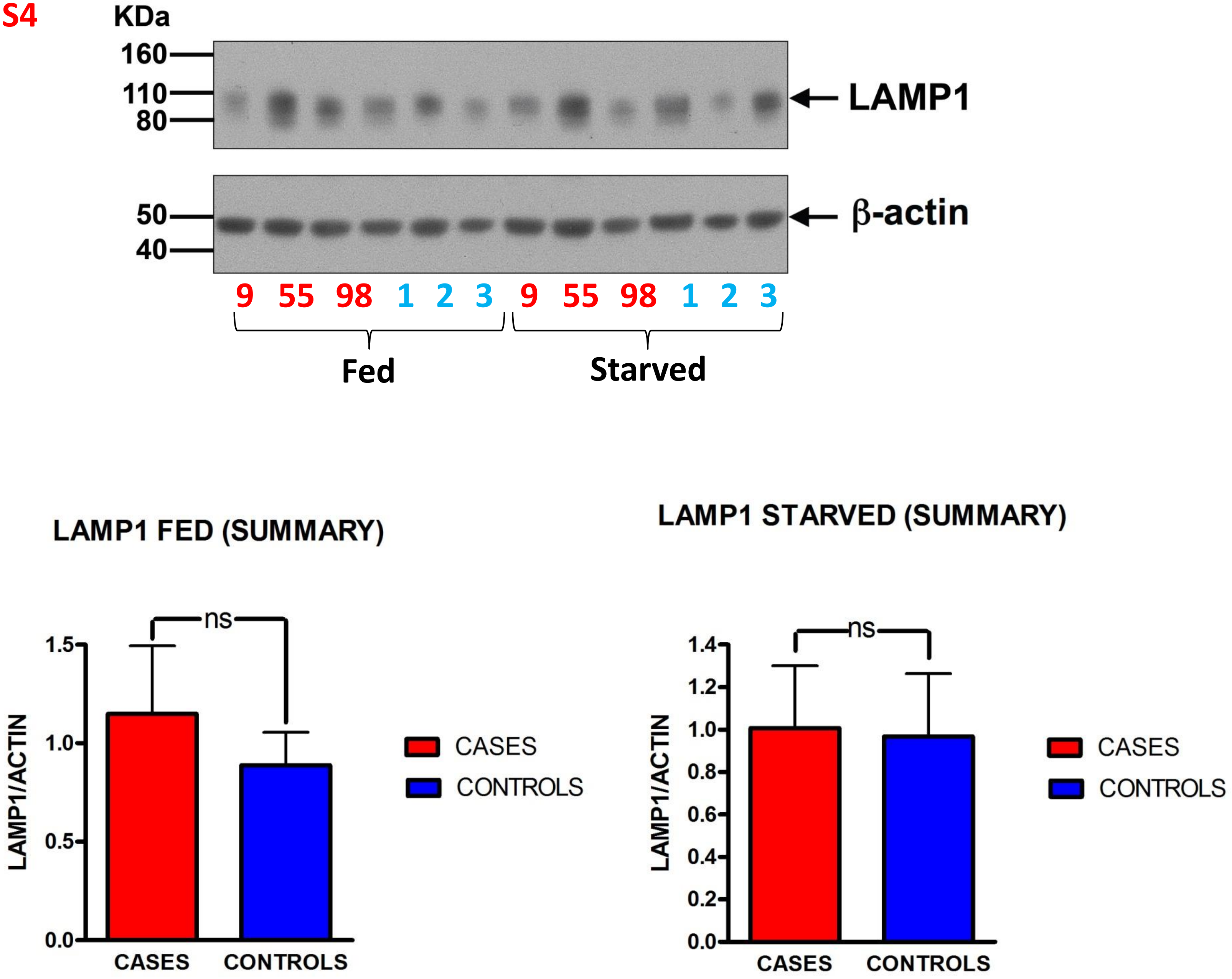
KDa
160
110
80
LAMP1
50
40
β-actin
9 55 98 1 2 3 9 55 98 1 2 3
Fed
Starved
LAMP1 FED (SUMMARY)
ns
1.5
1.0
0.5
0.0
LAMP1/ACTIN
CASES
CONTROLS
CASES
CONTROLS
LAMP1 STARVED (SUMMARY)
ns
1.4
1.2
1.0
0.8
0.6
0.4
0.2
0.0
LAMP1/ACTIN
CASES
CONTROLS
CASES
CONTROLS

Figure S5

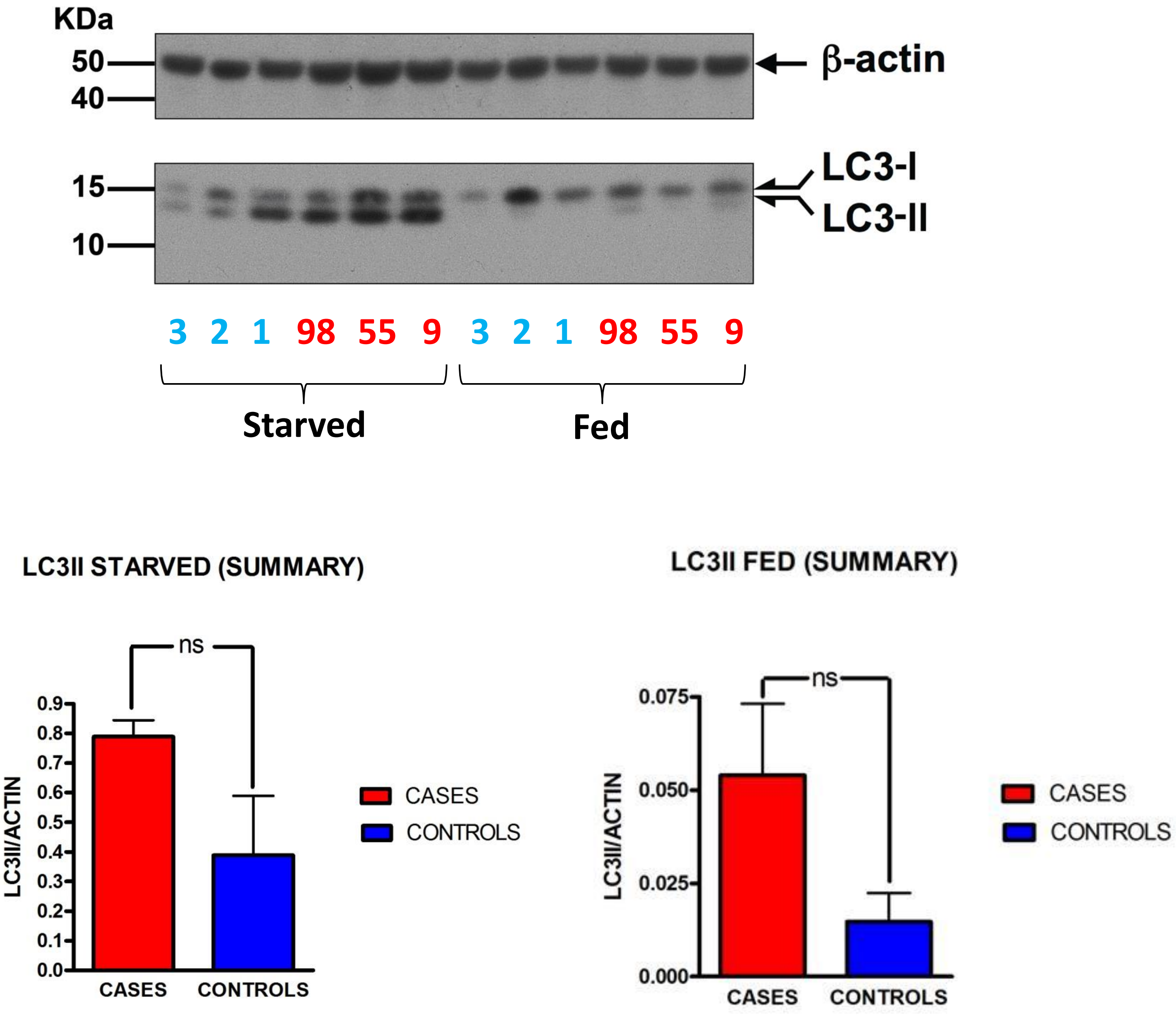
KDa
50
40
β-actin
15
10
LC3-I
LC3-II
3 2 1 98 55 9 3 2 1 98 55 9
Starved
Fed
LC3II STARVED (SUMMARY)
ns
LC3II/ACTIN
0.9
0.8
0.7
0.6
0.5
0.4
0.3
0.2
0.1
0.0
CASES
CONTROLS
CASES
CONTROLS
LC3II FED (SUMMARY)
ns
LC3II/ACTIN
0.075
0.050
0.025
0.000
CASES
CONTROLS
CASES
CONTROLS

Figure S6

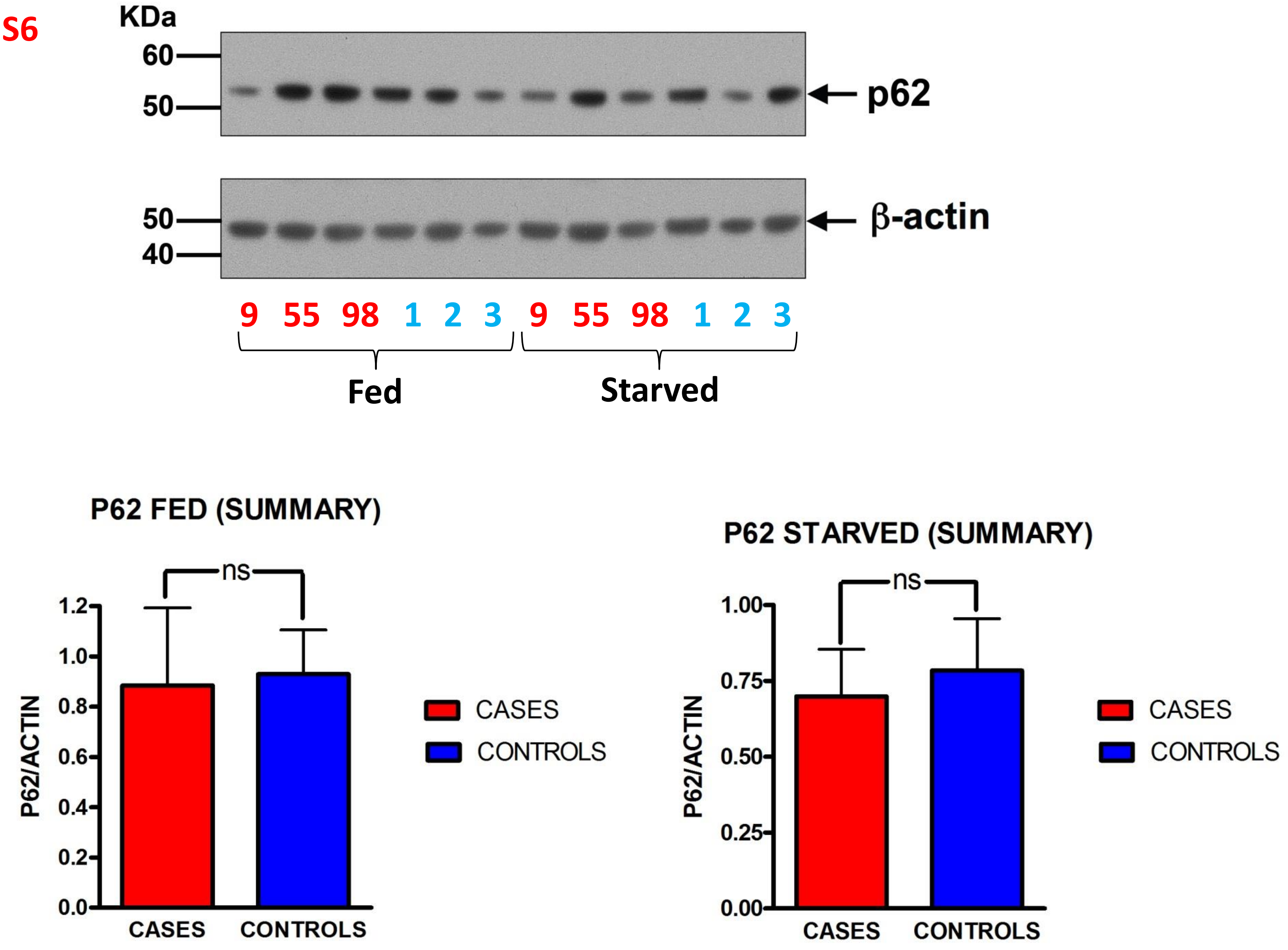
KDa
60
50
p62
50
40
β-actin
9 55 98 1 2 3 9 55 98 1 2 3
Fed
Starved
P62 FED (SUMMARY)
ns
P62/ACTIN
1.2
1.0
0.8
0.6
0.4
0.2
0.0
CASES
CONTROLS
CASES
CONTROLS
P62 STARVED (SUMMARY)
ns
P62/ACTIN
1.00
0.75
0.50
0.25
0.00
CASES
CONTROLS
CASES
CONTROLS

Figure S7

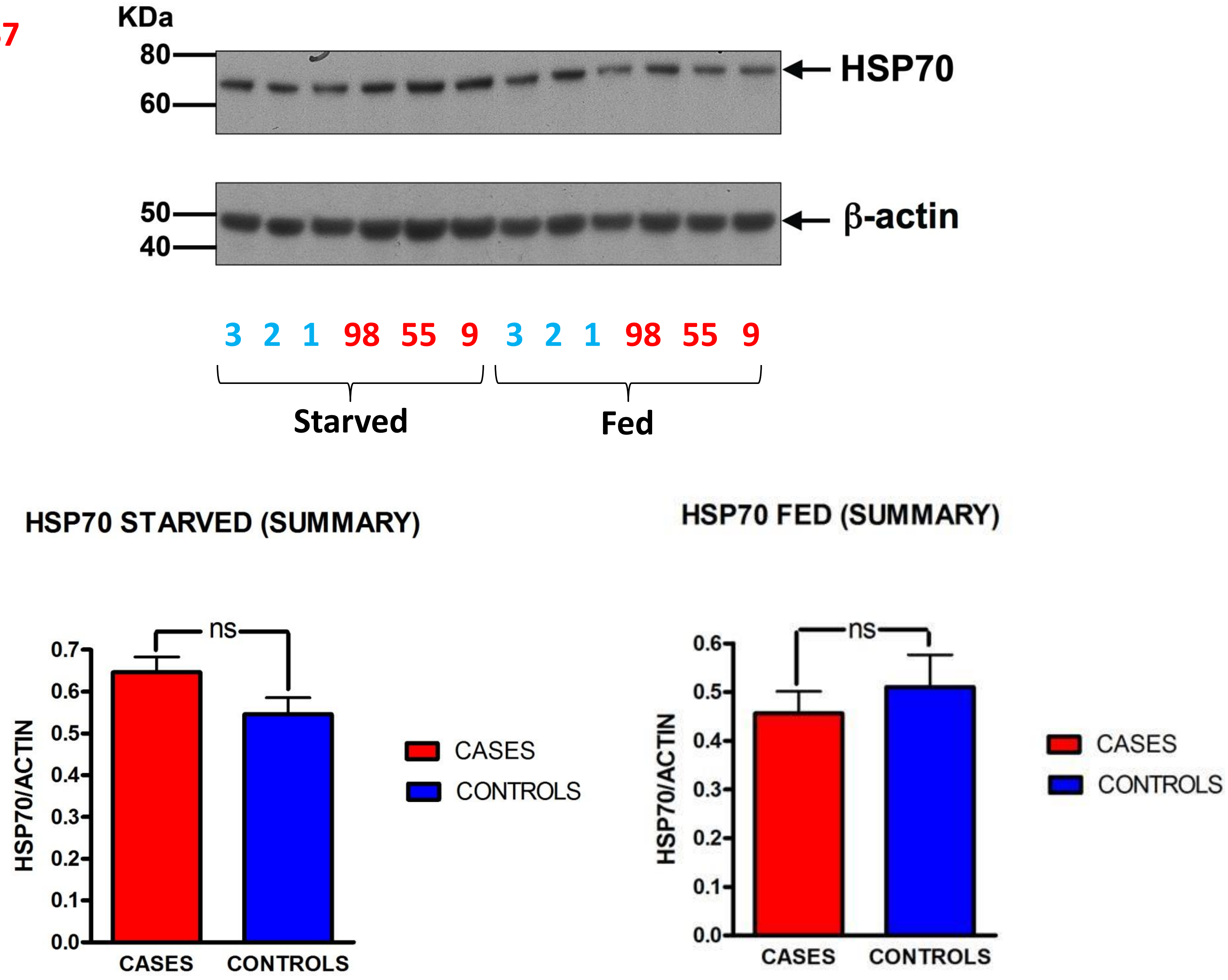
KDa
80
60
HSP70
50
40
β-actin
3 2 1 98 55 9 3 2 1 98 55 9
Starved
Fed
HSP70 STARVED (SUMMARY)
HSP70 FED (SUMMARY)
HSP70/ACTIN
0.0
0.1
0.2
0.3
0.4
0.5
0.6
0.7
ns
CASES
CONTROLS
