# Supplementary material for: Genetic and phenotypic characterization of complex hereditary spastic paraplegia
Source: Brain. 2016 May 23;139(7):1904–18. doi: 10.1093/brain/aww111 (PMC4939695; doi:10.1093/brain/aww111)
Supplement: Supplementary Data [file aww111_supplementary_data.zip › brain-2015-01890-File004.pdf]

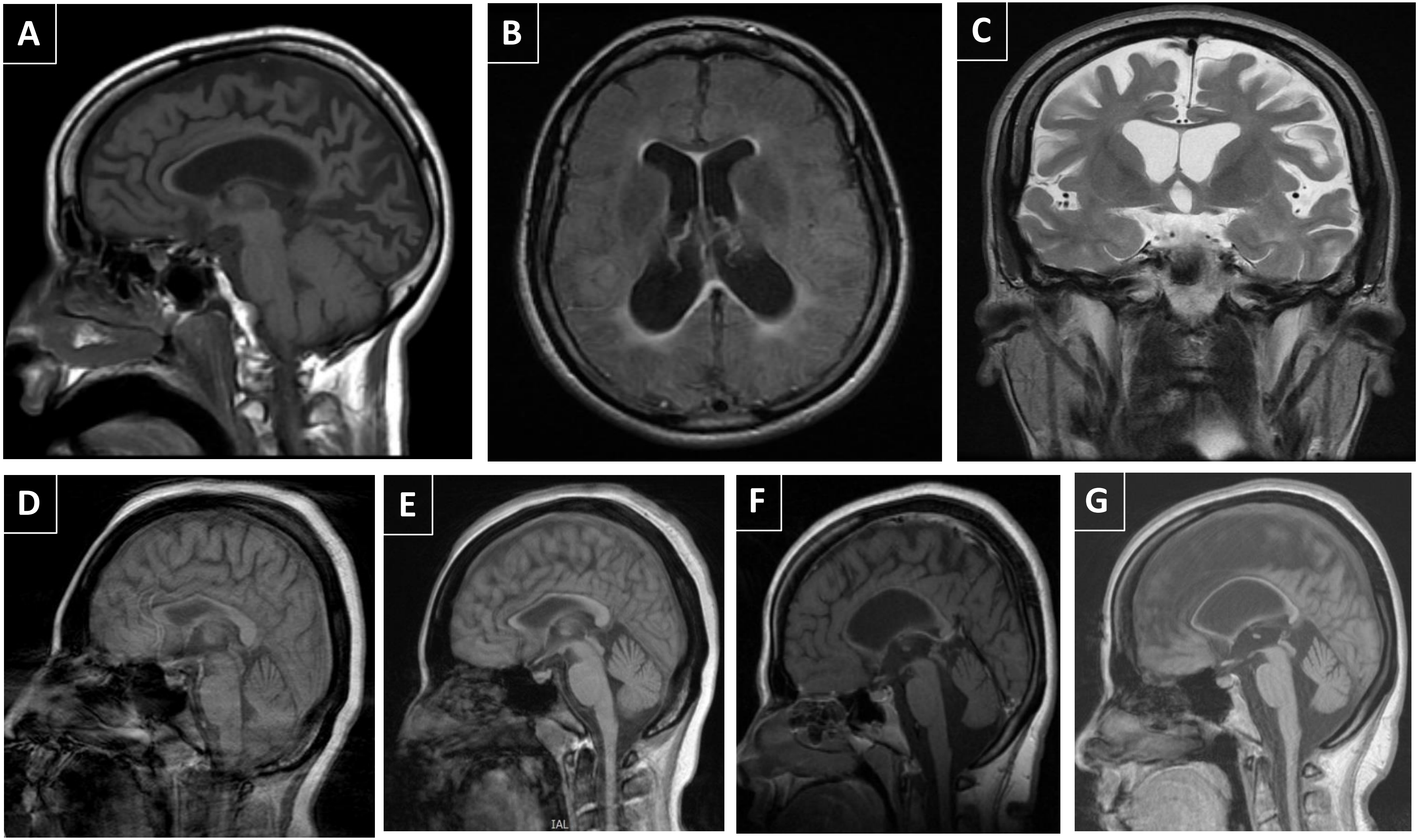

Supplementary MRI figure S1. Figure A to C. MRI of case 43 (age 26 years) with TPP1 and severe thinning of the corpus callosum and cerebral atrophy along with peri-venticular white matter abnormalities. Figure D and E are from case 10 with severe SPG11 at age 30 and 39 years and figure F and G are from case 16 with severe SPG11 at age 23 and 32 showing that when disease is severe the corpus callosum does not change significantly in the two cases here imaged over several years.

Supplementary figure S2

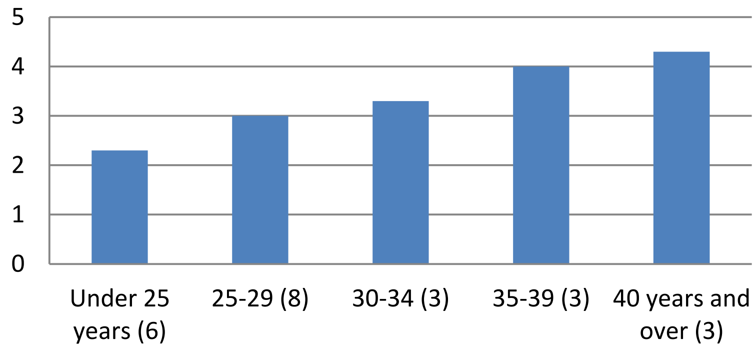

Figure S2. Modified Rankin score showing the level of disability of SPG11 patients with age. Number of cases in brackets .

Supplementary figure S3

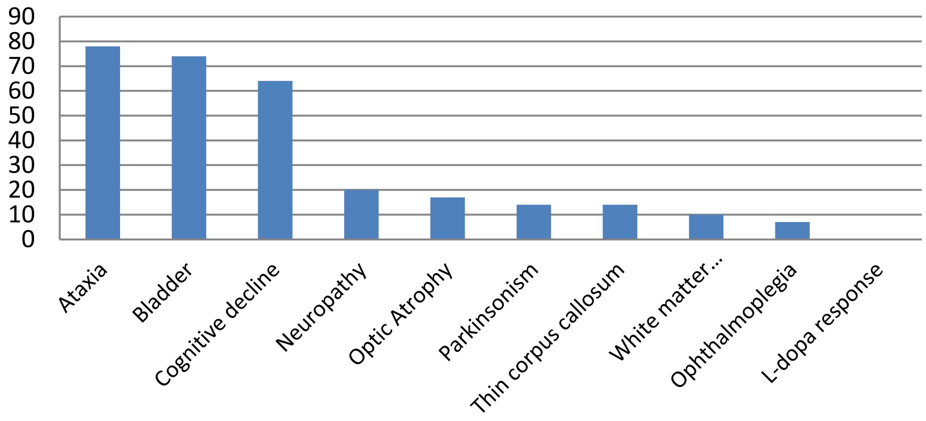

Figure S3. The frequency of clinical features that were present in addition to the spastic paraplegia in the negative probands sequenced.
