# Supplementary material for: Genetic and phenotypic characterization of complex hereditary spastic paraplegia
Source: Brain. 2016 May 23;139(7):1904–18. doi: 10.1093/brain/aww111 (PMC4939695; doi:10.1093/brain/aww111)
Supplement: Supplementary Data [file aww111_supplementary_data.zip › brain-2015-01890-File009.pdf]

<u>Supplemental text, tables and methods</u>

Variants of unknown significance were also identified (table S3) and discussed below.

These included cases with homozygous missense variants in *REEP1*, *SETX*, *SPTBN5* that were not present in our Northern European control brain series, in-house disease exome and were seen in the ExAC database of over 100,000 population cases in 15, 16 and 0 times as heterozygotes and never as homozygotes. The *REEP1* mutation is of particular interest as throughout this gene and codon there are heterozygous mutations associated with pure spastic paraplegia and it would be plausible, although not proven cause of complex HSP (Beetz *et al.* , 2008). The *CA8* homozygote was seen in three in-house and 21 ExAC heterozygous but not in the homozygous state and although this case was sent up as spastic ataxia there were no family history, family follow-up or functional work carried out to prove the pathogenicity. The *SPG11* A59V homozygote is also of unknown significance given the ExAC frequency. These cases did not have the full clinical characteristics of previously reported cases but could nevertheless represent a broadening of the clinical phenotype. We also identified a *GCH1* heterozygous mutation (Pro69Leu) that was previously reported as pathogenic in dopa-responsive dystonia (Clot *et al.* , 2009). This mutation was seen in the ExAC database at a frequency of 39 heterozygotes in over 100,000 cases and has also reported likely benign (Mencacci *et al.* , 2014) to indicate the effect of this mutation is still unknown. The same is true for the LRRK2 mutations which are present on the ExAC database and the associated patients do not have parkinsonian signs. Compound heterozygous mutations in the *PSAP* gene (c.C10G, p.L4V; c.G128A, p.G43E) were identified in family 40. The proband presented with early-onset spastic paraplegia complicated by myoclonic jerks, ataxia, cognitive decline and subtle eye movement problems. Her sister also developed a similar syndrome. The pathogenicity of the *PSAP* mutations in this family is still unknown as they would be unusual for the phenotype and are present in the ExAC database in a heterozygous state at a frequency of 42 and 75 in over 100,000 population individuals and the p.G43E present on two occasions as a homozygote. Variants in unreported genes, that are present at a rate of >10 heterozygotes on ExAC are of uncertain pathogenicity. In addition where the phenotype is unclear or unusual and no segregation is possible, functional work should be advocated to prove these defects. In the remaining

cases (51%) with complex HSP, a genetic defect was not identified. These probands had a mean age at onset of 16.7 years with complex HSP and the usual additional features` of ataxia, dystonia or cognitive decline.

**Table S4, *SPG11* PCR primer sequences used for PCR and sequencing**			
**1F**	AGTTAAGCAGAGTGGGAC	21F	TCATTAGAGAGTTCCATGTG
**1R**	TGAATAATGAAAGAATCAGC	21R	AAGTGATCCTCCTGCTTC
**2F**	ACTTATTCAACCCTTCTACC	22F	CTGATTTAATTCCATTTGC
**2R**	ACCTCTATGACTTTTCCTG	22R	AAACACTCACAATTCAATG
**3F**	ATCAAATCAAATGTACCAG	23F	CTATTCAAACAGAAATGCTC
**3R**	TTCCTATATCCCAGCTCC	23R	ACACCAGAGTTGTTCAAAG
**4F**	ACTTCATAGTCATCTGGTTG	24F	TTTCCAGTTCAAGCTAAAG
**4R**	TAGCCAGTGGTCAGTTATAC	24R	AGGCCTATGTCATGTCTAC
**5F**	TTAACAGGAGCAGTAGTAAC	25F	CTCTAACATTTTCAGCTCC
**5R**	TTTAATGAAAGGGTACAGC	25R	ATTATCAGCCTTTCCTGTC
**6F**	TTTTAAGAACATCTTTGCC	26F	CAGGAAATATCCATCTGTAG
**6R**	GAGGCAGAAGTAAAATACAG	26R	CATCATTATCTGTTGTTGG
**7F**	AAAACCATGATGAGTTAAG	27F	TATTTGGCATTATTCATTG
**7R**	TAAGCAGAGTTAGGGTAATG	27R	CGAGTGAGACACCAAGTC
**8F**	AGTCTCACCACATTAAATTC	28F	TTTCTTAGAGGTTCCCAG
**8R**	ATGACTAAGTTTTGGCAAG	28R	AATTTCCTAACTACCCCTC
**9F**	AATAAGACATTGGTCCTTG	29F	TTTTCCTTTAACCACATTAC
**9R**	TACCCAAATGTAGTAAATGG	29R	CAGATCAAGACCATCCTG
**10F**	TACCAAGTTTGAATTTTGAC	30F	GATATACTTAGGGAACATTAGC
**10R**	GAATTCTGTTTCTTTCTATTG	30-1F	AACTTGGAGAAGGAAAAC
**11F**	AAATGTATAATCCCATGTTG	30-1R	GAGCCATAGAGAGCTAGG
**11R**	AATGTCATTATTTCTTAGTGTC	30-2F	GAAATCAGGAGGAAACAG
**12F**	AGGGTGTTTCTGTATCTAAC	30-2R	CTTCCTTCTTGGAGAXAG
**12R**	TCTTCCAAGGTTTTCTTC	31F	ATTTCCTGGAAGAGGGCAAT
**13F**	AATAATCTTTAATTCCCAGC	31R	TTATCATCTAAAAGGCTGAC
**13R**	AGTTCCACATAAGAAACTTG	32F	CTGCTGAGGGTGAGGAGTCT
**14F**	AAAAGAGTGGATGTTCTTG	32R	TGCAATCCAGAAACTTGAGAGA
**14R**	AATATTATTTCCCGAAAGG	33F	AAGGGTTTCAAGCTCAGCAA
**15F**	CACAGCGAGATCCTGTCTCA	33R	TAGGCATCCAGAGCAGGAAC
**15R**	GGATTTATGGCATTTCAAAGGA	34F	CTTTGAGGATTGTGCCATGA
**16F**	TTTTAAGTAATTGAGACCTAAAC	34R	GCCCAGCCAACTCTCAAGTA
**16R**	AAAGTCACATTCAGGAGTC	35F	GGCATCTGAAAGCAACCACT
**17F**	TTAAATGCTAATCATCGC	35R	CTTGGGGAGGTCCCTAATTC
**17R**	TTCACAAGTTTAATACCATTC	36F	TTTAAGGTTTCTCCCTTTAG
**18F**	TCTCTCAGTTCATTGTTACC	36R	ACATAGTCAAACCCCATC
**18R**	TTTAAATTCAGCCTTATCC	37-38F	CTATTCTAGCCACGATCAC
**19F**	ATCTTGTTTCACAAGGTTC	37-38R	GACCTTACCTCTGGGTTC
**19R**	GAAAGATCTAGAGTGATTTCTG	39F	TTGTTTTCTACAACAGTTTG
**20F**	GAGCAGACTACCTCTGAAG	39R	TTAGCCATAAAATTCTTACAC
**20R**	GAAAACTAGATTGGCATTAC	40F	TGGGCAACAGAGTAAGAC
**21F**	TCATTAGAGAGTTCCATGTG	40R	GCTGTCCTGAGGAAGAGGAA
**21R**	AAGTGATCCTCCTGCTTC		
**22F**	CTGATTTAATTCCATTTGC		
**22R**	AAACACTCACAATTCAATG		
**23F**	CTATTCAAACAGAAATGCTC		
**23R**	ACACCAGAGTTGTTCAAAG		

Table S5, *SPG11* PCR program		
**Temperature (^{o}C)**	**Time (min)**	**Number of cycles**
**94**	01:00	
**94**	00:30	x15
**58**	00:30	
**72**	00:30	
**94**	00:30	x16
**58***	00:30	
**72**	00:30	
**94**	00:30	x14
**52**	00:30	
**72**	00:30	
**72**	05:00	

*Reducing temperature in each cycle.

Table S6, *SPG11* and control fibroblast cell lines used for biochemical studies

**Family /case**	**Gender**	**Age at biopsy (years)**	**Mutation**	**Disease status**	**Study**
9	F	40	c.6658_6659delAT, p.Met2220Aspfs*27 homozygous	Affected	Pilot study
22	F	31	c.6891_6893delGAT, p.I2298del / c.4237delinsTA, p.Val1413TyrFs*14	Affected	Pilot study
6a	F	32	c.5769delT, p.S1923RfsX28 homozygous	Affected	Pilot study
A	F	53	N/A	Unaffected	Pilot study
B	F	14	N/A	Unaffected	Pilot study
C	F	54	N/A	Unaffected	Pilot study
21	M	27	c.315delC, p.A106fsX121 homozygous	Affected	Replication study
5	M	19	c.3809T>A, p.V1270D homozygous	Affected	Replication study
23	F	25	c.2834 +1G>T, c.6754+4insTG	Affected	Replication study
6b	M	30	c.5769delT, p.S1923RfsX28 homozygous	Affected	Replication study
6c	F	26	c.5769delT, p.S1923RfsX28 homozygous	Affected	Replication study
D	M	9	N/A	Unaffected	Replication study
E	M	5	N/A	Unaffected	Replication study
F	M	44	N/A	Unaffected	Replication study
G	F	45	N/A	Unaffected	Replication study
H	F	25	N/A	Unaffected	Replication study
I	F	39	N/A	Unaffected	Replication study

Table S6, *SPG11* and control fibroblast cell lines used. The *SPG11* cases are from table 1. In family 6 three different affected patients were analysed. Unaffected carrier parents were not analysed. Controls are labelled A to I.

Table S7, *SPG11* exon 7-8 primers for cDNA PCR	
Forward	CTCATGATCCATGGAAGTGC
Reverse	CTGTGTCCAGCTGACGATTT
***GAPDH* exon 1-2 primers for cDNA PCR**	
Forward	AAGGTGAAGGTCGGAGTCAA
Reverse	AATGAAGGGGTCATTGATGG

Table S8, cDNA PCR cycling		
**Temperature (^{o}C)**	**Time (min)**	**Number of cycles**
**94**	00:20	X1
**94**	00:10	X45
**60**	00:20	
**4**	hold	

Supplementary methods: *Studies on patient-derived fibroblasts*

Autophagy was assessed through Western blot analysis of autophagy and lysosomal markers including LAMP1, LC3, p62, HSP70 as previously described (Manzoni *et al.* , 2013, Manzoni *et al.* , 2013). Fibroblasts were analysed in 2 separate studies: one pilot study including 3 cases and 3 controls, and one replication study including 5 cases and 6 controls. Three different treatments were completed. For the first treatment (long starvation), the growing medium was replaced with DMEM without FBS for 15h, followed by Earle's balanced salt solution (Sigma-Aldrich, UK) for 2.5h. For the second protocol (short starvation), the growing medium was replaced by Earle's balanced salt solution for 2.5h. For the third protocol, fibroblasts, after long starvation, were supplemented with MEM Amino Acids Solution (life technologies, UK) for 30min before harvesting. Cells were harvested on ice and lysated in RIPA buffer including protease (Roche) and phosphatase inhibitors (Pierce). The following antibodies were used for Western blotting: LC3 (NB100-2220, Novus Biologicals), LAMP1 (H4A3 Abcam), p62 (610833, BD Transduction Laboratories), β-actin (A1978, Sigma Aldrich), p70S6K total (sc-8418, Santa Cruz), phospho Thr389 P70S6K (sc-11759, Santa Cruz) and HSP70 (including reactivity to Hsc70, ab2787, AbCam). Each experiment was repeated at least three times. We compared intensity of LAMP1, HSP70, p62 and LC3II between cases and controls using the t-test. We also compared the ratio of LC3II/LC3I between cases and controls using the t-test. Statistical analyses were completed with Stata (StataCorp, 2011).

References

Beetz C, Schule R, Deconinck T, Tran-Viet KN, Zhu H, Kremer BP, et al. REEP1 mutation spectrum and genotype/phenotype correlation in hereditary spastic paraplegia type 31. Brain : a journal of neurology. 2008 Apr;131(Pt 4):1078-86.

Clot F, Grabli D, Cazeneuve C, Roze E, Castelnau P, Chabrol B, et al. Exhaustive analysis of BH4 and dopamine biosynthesis genes in patients with Dopa-responsive dystonia. Brain : a journal of neurology. 2009 Jul;132(Pt 7):1753-63.

Mencacci NE, Isaias IU, Reich MM, Ganos C, Plagnol V, Polke JM, et al. Parkinson's disease in GTP cyclohydrolase 1 mutation carriers. Brain : a journal of neurology. 2014 Sep;137(Pt 9):2480-92.

Manzoni C, Mamais A, Dihanich S, McGoldrick P, Devine MJ, Zerle J, et al. Pathogenic Parkinson's disease mutations across the functional domains of LRRK2 alter the autophagic/lysosomal response to starvation. Biochemical and biophysical research communications. 2013 Nov 6.

Manzoni C, Mamais A, Dihanich S, Abeti R, Soutar MP, Plun-Favreau H, et al. Inhibition of LRRK2 kinase activity stimulates macroautophagy. Biochimica et biophysica acta. 2013 Dec;1833(12):2900-10.

StataCorp. Stata Statistical Software: Release 12. College Station, TX: StataCorp LP. 2011.
