# Supplementary material for: Genetic and phenotypic characterization of complex hereditary spastic paraplegia
Source: Brain. 2016 May 23;139(7):1904–18. doi: 10.1093/brain/aww111 (PMC4939695; doi:10.1093/brain/aww111)
Supplement: Supplementary Data [file aww111_supplementary_data.zip › brain-2015-01890-File008.pdf]

Case	Gene	Variant	Variant type	Transcript	ExAC database	Sex	AAO (years)	Family History	Ethnic origin	Clinical Phenotype
56	SPTBN2	c.2305C>G, p.L769V	Homozygous	NM_006946.2	0/0	F	12	Yes	Egypt	Complex disorder with neuropathy, spasticity, ataxia, breathing problems, white matter abnormalities and intellectual loss.
57	SETX	c.T4150A, p.S1384T	Homozygous	NM_015046.5	16/0	M	5	Yes	Pakistan	Complex HSP with ataxia and epilepsy
58	KIF1A	c.1038G>C, p.R346S	Heterozygous	NM_001244008.1	0/0	F	Early 20s	No	UK	HSP with severe optic atrophy
59	REEP1	c.385C>T, p.R129C	Homozygous	NM_001164732.1	15/0	M	Childhood	No	Kenya	HSP with distal amyotrophy
60	GCH1	c.C206T, p.P69L	Heterozygous	NM_000161.2	39/0	F	18	No	UK	HSP, white matter abnormalities and ataxia
61	CA8	c.C479T, p.P160L	Homozygous	NM_004056.4	21/0	M	Teens	N/A	Asian	Spastic ataxia
62	LRRK2	c.C4612T, p.R1538C	Heterozygous	NM_198578.3	6/0	F	Early 20s	No	Nigeria	HSP, ataxia, dystonia, neuropathy
63	LRRK2	c. 4111A>G, p.I1371V	Heterozygous	NM_198578.3	126/1	F	Childhood	No	Pakistan	HSP and axonal neuropathy
64	LRRK2	c.5789C>G, p.P1930R	Heterozygous	NM_198578.3	0/0	F	Early 30s	No	UK	HSP and cognitive problems
40	PSAP	c.10C>G, p.L4V; c.128G>A, p.G43E	Compound heterozygous	NM_002778.2	42/0; 75/2	F	4	Yes	India	Complex HSP with seizures, ataxia and bradykinesia
31	SPG11	c.176C>T, p.A59V	Homozygous	NM_025137.3	157/1	F	7	Yes	India	HSP, ataxia and dystonia
42	KIF5A	c.2272G>A, p.E758K	Heterozygous	NM_004984.2	67/0	F	27	Yes	UK	HSP and cognitive. Scoiliosis and white matter abnormalities on MRI

Supplementary table S3. Genetic variants of unknown significance where the variation does not fit the clinical phenotype, inheritance pattern or a high frequency is seen on the ExAC database and segregation or functional studies on these changes have not been possible. See text for discussion on pathogenicity.
